# Social compatibility in opposite-sex prairie vole pairs is modulated by early-life sleep experience

**DOI:** 10.1371/journal.pbio.3003434

**Published:** 2026-03-27

**Authors:** Lezio S. Bueno-Junior, Noah E. P. Milman, Carolyn E. Jones-Tinsley, Anjesh Ghimire, Peyton T. Wickham, Yujia Hu, Bing Ye, Miranda M. Lim, Brendon O. Watson

**Affiliations:** 1 Department of Psychiatry, University of Michigan Medical School, Ann Arbor, Michigan, United States of America; 2 Portland VA Research Foundation, Portland, Oregon, United States of America; 3 Department of Neurology, Oregon Health and Science University, Portland, Oregon, United States of America; 4 Life Sciences Institute and Department of Cell and Developmental Biology, University of Michigan, Ann Arbor, Michigan, United States of America; 5 Imaging Core, Cleveland Clinic Research, Cleveland, Ohio, United States of America; The University of Edinburgh, UNITED KINGDOM OF GREAT BRITAIN AND NORTHERN IRELAND

## Abstract

Studies of human social behavior indicate stronger social affinity in matched-neurotype dyads (e.g., two individuals with autism or two without) compared to mixed-neurotype dyads (e.g., one individual with autism paired with one without). Is this dyad matching phenomenon also quantifiable in nonhuman animals? Using deep learning tools, we analyzed dyadic male-female interactions in prairie voles, a socially monogamous rodent species. To simulate “neurotypes”, voles were exposed to either control conditions or early-life sleep disruption (ELSD) during a critical neurodevelopmental period (post-natal days 14–21), recapitulating the influence of developmental sleep quality on later-life social behavior. Analogous to human studies, voles showed signs of reduced social affinity in mixed dyads compared to matched dyads, including sex-specific changes in aggression and body orientation toward the conspecific. These findings advance our understanding of social affinity between potential partners, providing a framework for new studies in both animal models and humans.

## Abstract 2

Human behavioral studies indicate that people have stronger social affinity for people with matched 'neurotypes'. This study shows a similar phenomenon can be observed in prairie voles; voles have higher social affinity for the opposite sex when placed in matched, versus mixed neurotype dyads.

## Introduction

During social interactions, we may experience feelings of closeness and affinity or, conversely, awkwardness and lack of affinity. Affinity, often expressed by nonverbal cues, facilitates attachment in both peer (friend) and sexual relationships [1,2]. Researchers have sought to quantify these feelings of “social chemistry” in humans, often through questionnaires to assess subjective perceptions about their social peers [3–10]. There have also been attempts to track these social properties more objectively in humans by measuring story recall, speech fluency, motion energy, facial expressions, mutual gaze, task cooperation, and inter-brain synchrony [11–24].

Most of these human studies have focused on the smallest social unit—the dyad—to assess how the behavioral similarity between individuals affects social affinity, or rapport. A common strategy in these studies is to form dyads with matched or mixed “neurotypes” or “behavioral styles”. Primarily, these studies have focused on autism spectrum disorders (ASD), including matched dyads (neurotypical-neurotypical or ASD-ASD) or mixed dyads (neurotypical-ASD or vice-versa), but dyad matching designs have also been used to study dysphoria, attention deficit, and collaborative decision-making [3–14,17,19,21–23]. Interestingly, across these studies, mixed dyads have generally been associated with lower social affinity than matched dyads. This aligns with the growing awareness that social difficulties may partly result from interpersonal mismatches, rather than solely from individual traits [25–28].

To explore dyad matching biologically, we can use rodent models. However, dyad matching research has limited correspondence in the rodent model literature, despite the existence of established rodent models in social neuroscience, as well as computational ethology tools for tracking groups of animals. Rodent models include social difference phenotypes or genotypes (e.g., prenatal valproic acid rat model and Shank3 knockout mouse model) [29,30], which capture some components of altered social behavior but are often limited by the focus on just one animal per assay. In turn, computational ethology tools include whole-body or body-part keypoint trackers (e.g., JAABA, idtracker.ai, DeepLabCut, nd SLEAP) [31–34], which track multiple animals but not individual social roles. Our group has contributed innovations to both areas. For rodent modeling, we have described that early-life sleep disruption (ELSD) impairs adult pair bonding in a monogamous rodent species: the prairie vole (*Microtus ochrogaster*), mimicking the link between developmental sleep issues and later-life altered social behavior [35]. Unlike traditional laboratory mice or rats, prairie voles exhibit socially monogamous behaviors both in the wild and the laboratory [36,37], making them a powerful model system for manipulating affiliative behaviors, as in our ELSD study [35]. In turn, for computational ethology, we initially published a DeepLabCut-based system to measure body orientation and distance between two spatially-restricted prairie voles [38]. Then, more recently, we co-developed LabGym2: a postural motif tracker that identifies social roles (e.g., “*chasing*” versus “*being chased*”) in groups of freely-moving animals, including prairie vole dyads [39].

In the present study, we integrated these innovations to quantify social roles in matched versus mixed prairie vole dyads, drawing a parallel to neurotype matching studies in humans. Specifically, male and female prairie vole pups were exposed to control conditions or ELSD from post-natal days 14–21, a critical neurodevelopmental window. Upon reaching adulthood, these voles were paired into matched or mixed dyads. Behaviors were then analyzed using long-duration recordings, lasting several hours to days. We found that mixed dyads exhibit altered body orientation behaviors and higher incidence of aggressive bouts, comparable to the lower affinity found previously in mixed human dyads. Furthermore, each behavior in prairie voles varied by sex and followed a different timeline, adding both sex specificity and temporal resolution to the available knowledge from human dyad studies. These findings have implications for both the basic and clinical understanding of social affinity, in addition to offering an analytical framework for future studies in different species.

## Results

### Overview of animal pairing and behavioral analysis

We utilized two behavioral phenotypes: ELSD prairie voles and age-matched controls (Ctrl), including both sexes. This resulted in four dyad combinations: Ctrl-Ctrl, ELSD-ELSD, Ctrl-ELSD, and ELSD-Ctrl (first and second acronyms represent male and female, respectively). A similar dyadic design was used in a recent study using Shank2 knockout mice (an ASD-like model), though in same-sex dyads [40], unlike the present study.

**Fig 1:**
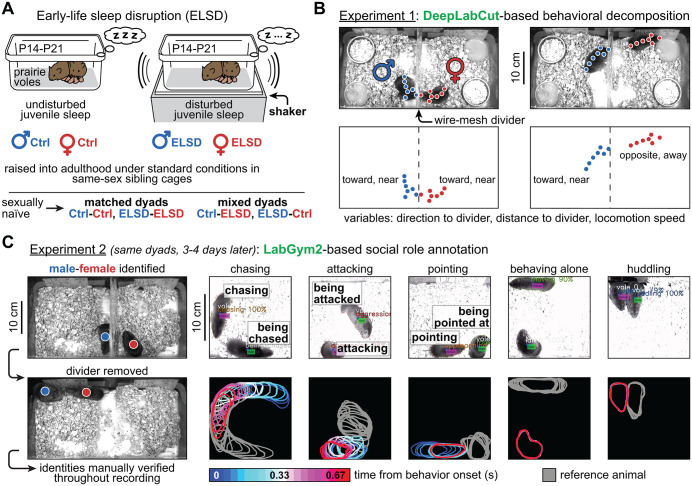
Overview of animal pairing and behavioral analysis. **A.** Early-life sleep disruption (ELSD) in prairie vole pups (post-natal days 14–21) generates “social difference” phenotypes, as indicated by behavioral and cytoarchitectural evidence [35,42]. ELSD subjects and their controls were raised into adulthood in same-sex sibling groups under standard conditions. The adults were then assigned into matched and mixed opposite-sex dyads. **B.** Experiment 1: male and female were filmed for 72 h while they interacted through a wire-mesh divider at the center of the cage. Cameras were positioned at an overhead angle and the animals were tracked using DeepLabCut [33], yielding cage-referenced behavioral variables. A similar method was utilized in a previous study [38] but without directly investigating dyad type. See also S1 Movie. **C.** Experiment 2: after 3–4 days of single housing in the vivarium, the same dyads were placed back together into the recording setup, this time for 4 h and without the divider. This resulted in a different kind of dataset comprising partner-referenced social role categories, annotated using LabGym2 [39] and manual validation (see Methods). The video snapshots (e.g., *chasing*, *attacking*) contain raw behavioral labels from LabGym2, as also shown in S2 Movie. Additional labels were included in this figure for clarity.

The ELSD method was initially validated by our group [35] and is illustrated in Fig 1A. Briefly, home cages containing both parents and their pups—post-natal days (P) 14–21—were placed on orbital shakers for gentle, intermittent agitation (see Methods). The pups were then weaned and housed with same-sex siblings under standard conditions until adulthood, generating cohorts of male and female-ELSD voles. Ctrl voles underwent the same conditions, except for sleep disruption with the orbital shaker (Fig 1A). Our previous studies [35,41] have shown that ELSD reduces rapid eye movement (REM) sleep time and fragments nonrapid eye movement (NREM) sleep in prairie vole pups while preserving their body weight, corticosterone levels, and parental care. Subsequent to these acute changes, we have shown that ELSD triggers long-term changes in social affiliation (e.g., partner-directed huddling during tests for pair bond formation), cognitive flexibility, object interactions, and neocortical cytoarchitecture [35,42,43].

Here, ESLD effects on dyadic behavior are explored more deeply thanks to computational ethology. We describe two strategies. Experiment 1: body-part keypoint tracking (DeepLabCut) [33] of mutually naïve male-female pairs separated by a wire-mesh divider (Fig 1B). Experiment 2: whole-body behavioral classification (LabGym2) [39] of the same male-female pairs, but without the divider (Fig 1C).

Experiment 1: initial divided cohabitation (Fig 1B and S1 Movie). This experiment consisted of 72-h overhead recordings where voles interacted through a wire-mesh divider at the center of the cage. This was the first encounter these animals had with each other. While this setup imposed a physical limitation on social interactions, it yielded uniform postural and spatial measures throughout recordings. This allowed us to decompose these measures into three DeepLabCut-derived variables: body direction to divider, distance to divider, and locomotion speed (Fig 1B). In a previous study [38], we characterized the sex-specific impacts of ELSD to these variables, including alterations in male body orientation and female circadian activity. However, we did not examine dyad matching effects in that study; such effects are reported here.

Experiment 2: subsequent undivided cohabitation (Fig 1C and S2 Movie). This experiment consisted of 4-h overhead recordings using the same voles, 3–4 days after Experiment 1, meaning that the opposite-sex mates had already been exposed to each other at the time of Experiment 2, i.e., dyad assignments were preserved between experiments (see Methods). In this setup, the animals were allowed to engage in freely-moving partner-referenced behaviors without a divider. This resulted in an enriched behavioral repertoire, which is more difficult to analyze with DeepLabCut but amenable to newer LabGym2-based behavioral classification (Fig 1C). One novel aspect of LabGym2—a software that we co-developed [39]—is the ability to categorize the social role of each individual (e.g., “*chasing*” versus “*being chased*”). We utilized this capability in combination with manual validation to ensure reliable identity tracking and sex specification per behavior. This resulted in the labeling of five behavior categories: *chasing*, *attacking*, *pointing*, *behaving alone*, and *huddling* (Fig 1C). Here, “*pointing*” combines sniffing behaviors with a freezing-like behavior that we often observed after each aggressive bout (see S2 Movie). See Methods for details.

Therefore, our two experiments yielded very different analyses. Nevertheless, dyad matching effects emerged in both experiments, as reported below.

### Experiment 1: Mixed dyads showed exacerbated sex differences in body orientation

**Fig 2:**
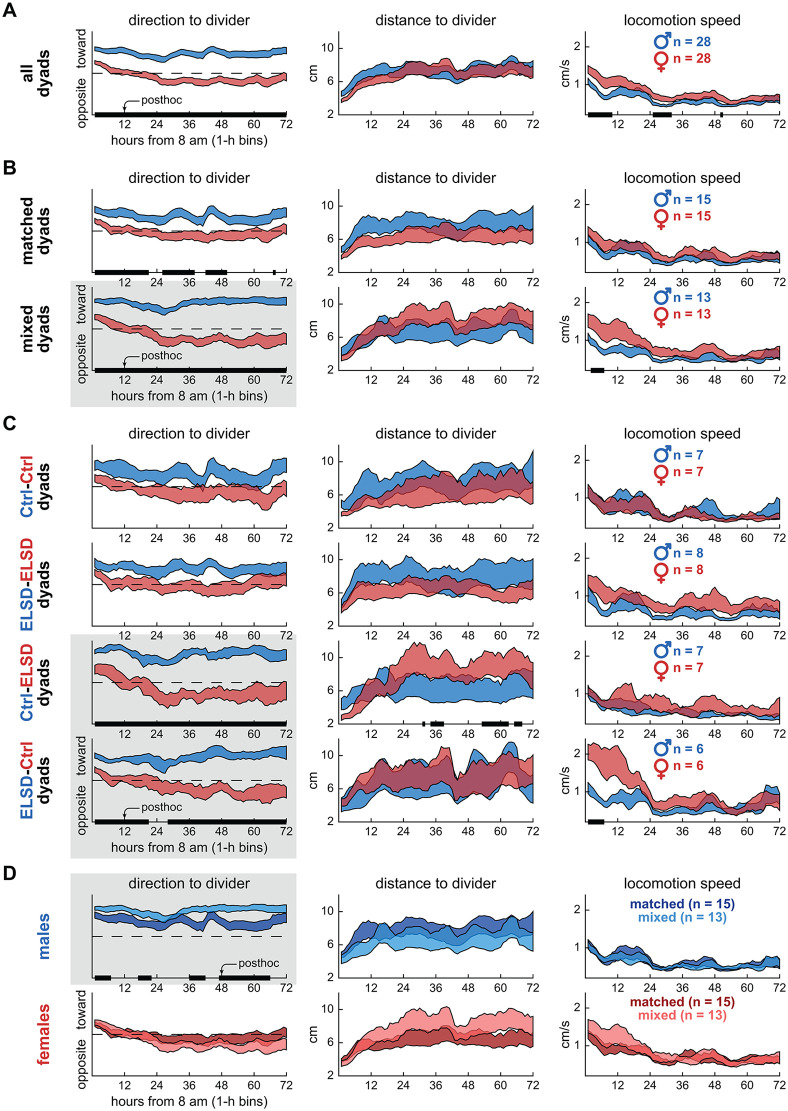
Experiment 1: Mixed dyads showed exacerbated sex differences in body orientation. **A.** Time series data (72 h, 1-h bins) showing sex differences in direction to divider across all dyads. Males spent more time toward the divider than females throughout the recordings. The curves represent mean ± standard error aggregated across animals, regardless of individual dyad assignments. The black bars on the x-axes represent post-hoc differences, after two-way ANOVA with time bins as repeated measures. **B.** Sex differences in direction to divider were more pronounced in mixed dyads (highlighted with gray background). Distance to divider and locomotion speed were less sensitive to dyad type, illustrating that behavioral decomposition was important to capture the effects shown here. **C.** Further specification into the four dyad subtypes. Sex differences in direction to divider were confined to the mixed-dyad subtypes (Ctrl-ELSD, ELSD-Ctrl). Additional smaller effects were observed in distance to divider and locomotion speed, specific to each mixed-dyad subtype. **D.** Same groupings as panel B but comparing matched and mixed dyads within each sex. The tendency to behave toward the divider was higher in males and further increased in mixed-dyad males. See also Table 1 for statistics, S1, S2, S5, and S6 Figs, and S1 Movie. Underlying processed data and plotting code for this figure are available at figshare (https://doi.org/10.6084/m9.figshare.31820266).

In Experiment 1, a male and female interacted through a wire-mesh divider. Fig 2 describes these interactions over 72 h (1-h bins, see x-axes), with graph columns displaying DeepLabCut-derived behavioral decomposition variables. Fig 2A starts by comparing sexes irrespective of dyad grouping. When analyzing direction to divider, we found that male voles spent significantly more time facing toward the divider than females throughout the recording (two-way ANOVA with repeated measures, effect of sex groups, F_1,27_ = 37.78, *P* < 0.001). For distance to divider, both sexes preferred areas near the divider (4–6 cm) during the initial 12 h of recording; this variable later stabilized at around 6 cm, halfway between divider and opposite cage wall (effect of time, F_71,1,917_ = 9.06, *P* < 0.001). For locomotion speed (Fig 2A), both sexes exhibited higher locomotion in the initial 12 h of recording; this variable later stabilized to lower basal levels (effect of time, F_71,1,917_ = 19.10, *P* < 0.001). These results corroborate our previous findings [38] that sex-specific body orientation patterns persist for at least 72 h in the divided cage, despite the stabilization of locomotion and area preference after the initial 12 h. See Table 1 for a comprehensive statistics panel and S1 Movie for representative behaviors.

**Table 1:** Statistics for Fig 2.

Figure panel	Dyad type or sex	Effect	Value	Direction to divider	Distance to divider	Locomotion speed
2A	All dyads	Sex	deg fr	1, 27	1, 27	1, 27
			F; *P*	**=37.78; <0.001**	=0.13; =0.722	=4.03; =0.055
		Time	deg fr	71, 1917	71, 1917	71, 1917
			F; *P*	=7.92; <0.001	=9.06; <0.001	=19.10; <0.001
		Interaction	deg fr	71, 1917	71, 1917	71, 1917
			F; *P*	**=3.13; <0.001**	=1.01; =0.460	**=1.73; <0.001**
2B	Matched dyads	Sex	deg fr	1, 14	1, 14	1, 14
			F; *P*	=7.72; =0.015	=1.50; =0.241	=0.85; =0.373
		Time	deg fr	71, 994	71, 994	71, 994
			F; *P*	=4.46; <0.001	=3.51; <0.001	=11.10; <0.001
		Interaction	deg fr	71, 994	71, 994	71, 994
			F; *P*	=1.06; =0.340	=0.73; =0.955	=0.76; =0.929
	Mixed dyads	Sex	deg fr	1, 12	1, 12	1, 12
			F; *P*	**=72.53; <0.001**	=1.30; =0.276	=4.52; =0.055
		Time	deg fr	71, 852	71, 852	71, 852
			F; *P*	=4.33; <0.001	=6.06; <0.001	=8.88; <0.001
		Interaction	deg fr	71, 852	71, 852	71, 852
			F; *P*	**=4.52; <0.001**	=1.05; =0.373	**=1.63; =0.001**
2C	Ctrl- Ctrl	Sex	deg fr	1, 6	1, 6	1, 6
			F; *P*	=3.04; =0.132	=0.39; =0.553	=0.15; =0.715
		Time	deg fr	71, 426	71, 426	71, 426
			F; *P*	=3.54; <0.001	=2.80; <0.001	=5.74; <0.001
		Interaction	deg fr	71, 426	71, 426	71, 426
			F; *P*	=0.77; =0.915	=0.49; =1.000	=0.41; =1.000
	ELSD- ELSD	Sex	deg fr	1, 7	1, 7	1, 7
			F; *P*	=4.27; =0.078	=1.17; =0.315	=1.71; =0.232
		Time	deg fr	71, 497	71, 497	71, 497
			F; *P*	=2.14; <0.001	=1.51; =0.007	=5.39; <0.001
		Interaction	deg fr	71, 497	71, 497	71, 497
			F; *P*	=0.89; =0.720	=0.61; =0.995	=1.00; =0.485
	Ctrl- ELSD	Sex	deg fr	1, 6	1, 6	1, 6
			F; *P*	**=35.70; <0.001**	=5.02; =0.066	=1.10; =0.335
		Time	deg fr	71, 426	71, 426	71, 426
			F; *P*	=3.86; <0.001	=3.78; <0.001	=3.13; <0.001
		Interaction	deg fr	71, 426	71, 426	71, 426
			F; *P*	**=2.31; <0.001**	**=2.57; <0.001**	=0.41; =1.000
	ELSD- Ctrl	Sex	deg fr	1, 5	1, 5	1, 5
			F; *P*	**=37.71; =0.002**	=0.05; =0.838	=3.62; =0.115
		Time	deg fr	71, 355	71, 355	71, 355
			F; *P*	=1.24; =0.111	=2.77; <0.001	=7.65; <0.001
		Interaction	deg fr	71, 355	71, 355	71, 355
			F; *P*	**=2.52; <0.001**	=0.35; =1.000	**=3.46; <0.001**
2D	Males	Dyad type	deg fr	1, 12	1, 12	1, 12
			F; *P*	=8.26; =0.014	=0.96; =0.346	=0.03; =0.865
		Time	deg fr	71, 852	71, 852	71, 852
			F; *P*	=3.15; <0.001	=3.44; <0.001	=8.36; <0.001
		Interaction	deg fr	71, 852	71, 852	71, 852
			F; *P*	**=1.58; =0.002**	=0.54; =0.999	=0.55; =0.999
	Females	Dyad type	deg fr	1, 12	1, 12	1, 12
			F; *P*	=1.42; =0.257	=2.05; =0.178	=0.28; =0.607
		Time	deg fr	71, 852	71, 852	71, 852
			F; *P*	=7.47; <0.001	=7.12; <0.001	=9.06; <0.001
		Interaction	deg fr	71, 852	71, 852	71, 852
			F; *P*	=1.09; =0.302	=0.97; =0.540	=1.30; =0.055

Degrees of freedom, F, and *P* values were obtained using two-way ANOVA with time bins as repeated measures. *P* values < 0.01 were highlighted with bold font, except for repeated measure effects.

By parsing these sex differences into dyad types, we begin to reveal the novel findings of this study. Fig 2B shows that while sex differences in direction to divider were significant in both matched and mixed dyads, these differences were more pronounced in mixed dyads and persisted for 72 h (effect of sex groups, F_1,12_ = 72.53, *P* < 0.001). Fig 2C further divides male and female voles into the four juvenile sleep status combinations. In this case, sex differences in direction to divider were significant only in mixed dyads: Ctrl-ELSD (effect of sex groups, F_1,6_ = 35.70, *P* < 0.001) and ELSD-Ctrl (effect of sex groups, F_1,5_ = 37.71, *P* = 0.002). Matched dyads, including Ctrl-Ctrl and ELSD-ELSD, showed no significant sex differences (Table 1). These results indicate that body orientation behaviors did not change linearly with the number of ELSD individuals in the dyad; rather, dyad matching was the main factor modulating body orientation.

Additional sex differences were specific to mixed dyads and confined to more localized time periods, as indicated by post-hoc differences in the distance to divider and locomotion speed graphs (Fig 2C). We observed that ELSD females in Ctrl-ELSD dyads maintained a greater distance from the divider more than 36 h into the session (effect of interaction, F_71,426_ = 2.57, *P* < 0.001), while Ctrl females in ELSD-Ctrl dyads exhibited higher locomotion speed during the initial 12 h compared to males (effect of interaction, F_71,355_ = 3.46, *P* < 0.001; Fig 2C). However, these sex differences were modest compared to the prominent results in direction to divider (Table 1). Fig 2D further highlights direction to divider as a sensitive variable, this time comparing matched and mixed dyads within each sex. Males spent significantly more time toward the divider in mixed compared to matched dyads (effect of interaction, F_71,852_ = 1.58, *P* = 0.002), indicating heightened female-directed orientation in mixed-dyad males (Table 1). See also: S1 and S2 Figs for supplementary details on behavioral bouts, S5 Fig for age distribution across sexes and dyad types (we found no evidence that age was a confounder in our data), and S6 Fig for dyad-based behavioral variables (e.g., mutual body direction and mutual distance).

Overall, Fig 2 shows that a supra-individual factor (dyad matching) preferentially modulated one behavioral component (body direction) in one sex (males). Next, we describe that dyad matching remained an important factor in a different environment.

### Experiment 2: Mixed dyads showed higher female-to-male aggression

**Fig 3:**
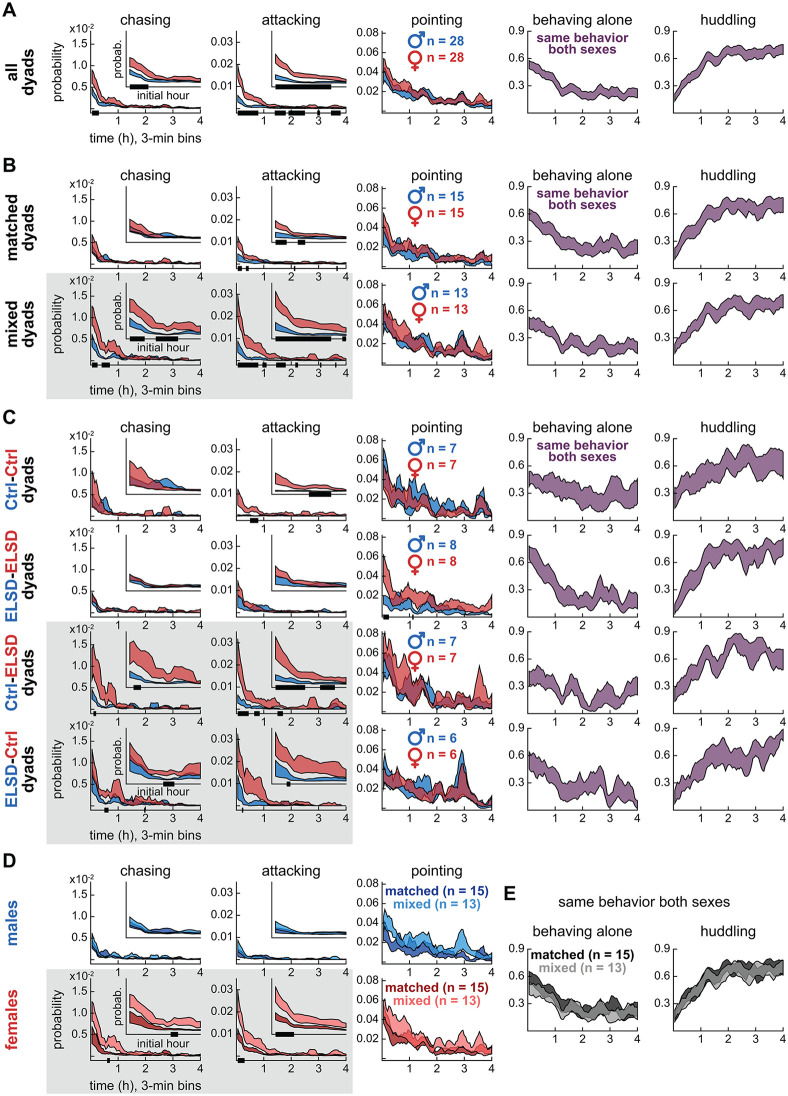
Experiment 2: Mixed dyads showed higher female-to-male aggression. **A.** Time series data (4 h, 3-min bins) showing sex differences in the likelihood of chasing and attacking across all dyads. Females were more likely to chase and attack than males, especially in the initial hour of recording. The curves represent mean ± standard error aggregated across animals, regardless of individual dyad assignments. The black bars on the x-axes represent post-hoc differences, after two-way ANOVA with time bins as repeated measures. Inset plots show probability within the first hour. **B.** Sex differences in the likelihood of *chasing* and *attacking* were more pronounced in mixed dyads (highlighted by gray background). *Pointing* behaviors, including face-to-face, face-to-body, and anogenital sniffing, showed no sex or dyad type specificity, making them useful comparators (see Methods for details). The role-unspecific categories of *behaving  alone* and *huddling* were more dominant and mutually exclusive, illustrating the transition from high activity (periods of *moving* and *idling alone* punctuated by *chasing*, *aggression*, and *sniffing* bouts) to low activity (periods of continuous *huddling*). **C.** Further specification into the four dyad subtypes. Sex differences in the likelihood of *chasing* and *attacking* were stronger in the mixed-dyad subtypes (Ctrl-ELSD, ELSD-Ctrl). **D.** Same groupings as panel B but comparing matched and mixed dyads within each sex. Mixed-dyad females were more likely to chase and attack than matched-dyad females. See also Table 2 for statistics, S3–S5 Figs, and S2 Movie. Underlying processed data and plotting code for this figure are available at figshare (https://doi.org/10.6084/m9.figshare.31820266).

In Experiment 2, the wire-mesh divider was not present, and animals were able to interact in an unrestricted fashion. Fig 3 is structured similarly to the results above, with two differences: the recordings lasted 4 h (3-min bins, see x-axes) and the graph columns show LabGym2-based behavior categories. We again start by comparing sexes irrespective of dyad grouping in Fig 3A. The social roles of *chasing* (two-way ANOVA with repeated measures, effect of interaction, F_79,2,133_ = 3.89, *P* < 0.001) and *attacking* (effect of interaction, F_79,2,133_ = 11.30, *P* < 0.001) were significantly more prevalent in female voles, especially in the first hour of recording (insets), after which these variables decayed to lower basal levels. The social role of *pointing*, which included sniffing and other behaviors (see Methods), was evenly distributed across sexes and showed a more gradual decline over the 4-h period (effect of time, F_79,2,133_ = 8.95, *P* < 0.001). The role-unspecific categories of *behaving alone* and *huddling* (i.e., behaviors that considered the full dyad, see Methods) also showed gradual time courses, but in opposite directions: *behaving alone* decreased (effect of time, F_79,2,133_ = 8.64, *P* < 0.001), while *huddling* proportionally increased (effect of time, F_79,2,133_ = 13.89, *P* < 0.001) over time (Fig 3A). In addition, *behaving alone* and *huddling* were the most frequent behaviors across Fig 3, as indicated by the probability axes. See Table 2 for a comprehensive statistics panel and S2 Movie for representative behaviors.

**Table 2:** Statistics for Fig 3.

Figure panel	Dyad typeor sex	Effect	Value	Chasing	Attacking	Pointing	Behaving alone	Huddling
3A	All dyads	Sex	deg fr	1, 27	1, 27	1, 27	1, 27	1, 27
			F; *P*	=4.79; =0.038	**=16.28; <0.001**	=1.11; =0.302	=0.00; =1.000	=0.00; =1.000
		Time	deg fr	79, 2,133	79, 2,133	79, 2,133	79, 2,133	79, 2,133
			F; *P*	=19.20; <0.001	=21.83; <0.001	=8.95; <0.001	=8.64; <0.001	=13.89; <0.001
		Interaction	deg fr	79, 2,133	79, 2,133	79, 2,133	79, 2,133	79, 2,133
			F; *P*	**=3.89; <0.001**	**=11.30; <0.001**	=0.95; =0.607	=0.00; =1.000	=0.00; =1.000
3B	Matched dyads	Sex	deg fr	1, 14	1, 14	1, 14	1, 14	1, 14
			F; *P*	=0.31; =0.588	=3.77; =0.073	=0.72; =0.409	=0.00; =1.000	=0.00; =1.000
		Time	deg fr	79, 1,106	79, 1,106	79, 1,106	79, 1,106	79, 1,106
			F; *P*	=8.72; <0.001	=12.96; <0.001	=4.54; <0.001	=6.06; <0.001	=8.66; <0.001
		Interaction	deg fr	79, 1,106	79, 1,106	79, 1,106	79, 1,106	79, 1,106
			F; *P*	=0.61; =0.997	**=4.25; <0.001**	=0.64; =0.993	=0.00; =1.000	=0.00; =1.000
	Mixed dyads	Sex	deg fr	1, 12	1, 12	1, 12	1, 12	1, 12
			F; *P*	=6.87; =0.022	**=15.96; =0.002**	=0.35; =0.565	=0.00; =1.000	=0.00; =1.000
		Time	deg fr	79, 948	79, 948	79, 948	79, 948	79, 948
			F; *P*	=12.11; <0.001	=13.83; <0.001	=4.77; <0.001	=3.08; <0.001	=5.38; <0.001
		Interaction	deg fr	79, 948	79, 948	79, 948	79, 948	79, 948
			F; *P*	**=5.25; <0.001**	**=8.37; <0.001**	=0.88; =0.757	=0.00; =1.000	=0.00; =1.000
3C	Ctrl- Ctrl	Sex	deg fr	1, 6	1, 6	1, 6	1, 6	1, 6
			F; *P*	=0.06; =0.822	=8.63; =0.026	=0.35; =0.578	=0.00; =1.000	=0.00; =1.000
		Time	deg fr	79, 474	79, 474	79, 474	79, 474	79, 474
			F; *P*	=4.49; <0.001	=3.82; <0.001	=2.33; <0.001	=1.42; =0.015	=2.75; <0.001
		Interaction	deg fr	79, 474	79, 474	79, 474	79, 474	79, 474
			F; *P*	=0.52; =1.000	**=3.58; <0.001**	=0.29; =1.000	=0.00; =1.000	=0.00; =1.000
	ELSD- ELSD	Sex	deg fr	1, 7	1, 7	1, 7	1, 7	1, 7
			F; *P*	=0.91; =0.373	=0.31; =0.598	=3.47; =0.105	=0.00; =1.000	=0.00; =1.000
		Time	deg fr	79, 553	79, 553	79, 553	79, 553	79, 553
			F; *P*	=6.16; <0.001	=9.99; <0.001	=2.48; <0.001	=6.15; <0.001	=6.72; <0.001
		Interaction	deg fr	79, 553	79, 553	79, 553	79, 553	79, 553
			F; *P*	=0.68; =0.981	**=1.45; =0.010**	**=1.76; <0.001**	=0.00; =1.000	=0.00; =1.000
	Ctrl- ELSD	Sex	deg fr	1, 6	1, 6	1, 6	1, 6	1, 6
			F; *P*	=3.54; =0.109	=9.11; =0.023	=0.67; =0.444	=0.00; =1.000	=0.00; =1.000
		Time	deg fr	79, 474	79, 474	79, 474	79, 474	79, 474
			F; *P*	=6.24; <0.001	=6.58; <0.001	=3.84; <0.001	=2.30; <0.001	=3.53; <0.001
		Interaction	deg fr	79, 474	79, 474	79, 474	79, 474	79, 474
			F; *P*	**=3.86; <0.001**	**=6.76; <0.001**	=0.65; =0.990	=0.00; =1.000	=0.00; =1.000
	ELSD- Ctrl	Sex	deg fr	1, 5	1, 5	1, 5	1, 5	1, 5
			F; *P*	=2.92; =0.148	=5.97; =0.058	=0.06; =0.816	=0.00; =1.000	=0.00; =1.000
		Time	deg fr	79, 395	79, 395	79, 395	79, 395	79, 395
			F; *P*	=5.91; <0.001	=7.17; <0.001	=1.96; <0.001	=1.91; <0.001	=2.85; <0.001
		Interaction	deg fr	79, 395	79, 395	79, 395	79, 395	79, 395
			F; *P*	**=2.20; <0.001**	**=3.28; <0.001**	=0.57; =0.999	=0.00; =1.000	=0.00; =1.000
3D	Males	Dyad type	deg fr	1, 12	1, 12	1, 12	1, 12	1, 12
			F; *P*	=0.60; =0.454	=0.01; =0.943	=1.72; =0.215	=0.67; =0.430	=0.11; =0.746
		Time	deg fr	79, 948	79, 948	79, 948	79, 948	79, 948
			F; *P*	=11.40; <0.001	=6.25; <0.001	=5.16; <0.001	=6.94; <0.001	=11.35; <0.001
		Interaction	deg fr	79, 948	79, 948	79, 948	79, 948	79, 948
			F; *P*	=0.90; =0.716	=0.82; =0.873	=0.66; =0.990	=0.45; =1.000	=0.35; =1.000
	Females	Dyad type	deg fr	1, 12	1, 12	1, 12	1, 12	1, 12
			F; *P*	**=10.81; =0.006**	=5.82; =0.033	=2.77; =0.122	=0.67; =0.430	=0.11; =0.746
		Time	deg fr	79, 948	79, 948	79, 948	79, 948	79, 948
			F; *P*	=13.05; <0.001	=19.21; <0.001	=8.67; <0.001	=6.935; <0.001	=11.35; <0.001
		Interaction	deg fr	79, 948	79, 948	79, 948	79, 948	79, 948
			F; *P*	**=2.70; <0.001**	**=4.42; <0.001**	=0.36; =1.000	=0.448; =1.000	0.35; =1.000

Degrees of freedom, F, and *P* values were obtained using two-way ANOVA with time bins as repeated measures. *P* values < 0.01 were highlighted with bold font, except for repeated measure effects.

Despite their lower occurrence rate, *chasing* and *attacking* are the behaviors with the greatest differential trajectories across types of dyads. As shown in Fig 3B, female voles were more likely to chase (effect of interaction, F_79,948_ = 5.25, *P* < 0.001) and attack (effect of interaction, F_79,948_ = 8.37, *P* < 0.001) the males in mixed than matched dyads (Table 2). Consistently, in dyad subgroups (Fig 3C), female-to-male *chasing* and *attacking* were more frequent in both Ctrl-ELSD (*chasing*, effect of interaction, F_79,474_ = 3.86, *P* < 0.001; *attacking*, effect of interaction, F_79,474_ = 6.76, *P* < 0.001) and ELSD-Ctrl dyads (*chasing*, effect of interaction, F_79,395_ = 2.20, *P* < 0.001; *attacking*, effect of interaction, F_79,395_ = 3.28, *P* < 0.001). Matched ELSD-ELSD dyads showed no sex differences in *chasing* and a comparatively small difference in *attacking* (effect of interaction, F_79,553_ = 1.45, *P* = 0.010), reinforcing that these behaviors did not change linearly with the number of ELSD individuals in the dyad, similar to the findings in dyads behaving across a divider. Additional sex differences were observed in *attacking* (Ctrl-Ctrl; effect of interaction, F_79,474_ = 3.58, *P* < 0.001) and *pointing* (ELSD-ELSD; effect of interaction, F_79,553_ = 1.76, *P* < 0.001; Fig 3C), but these were also modest compared to the sex differences in *chasing* and *attacking* behaviors found in mixed dyads (Table 2). Furthermore, Fig 3D shows within-sex comparisons between dyad types, confirming that mixed-dyad females were more likely to chase (effect of interaction, F_79,948_ = 2.70, *P* < 0.001) and attack (effect of interaction, F_79,948_ = 4.42, *P* < 0.001) than matched-dyad females. No dyad type differences were observed in the other behaviors, i.e., *pointing*, *behaving alone*, and *huddling* (Fig 3D and 3E and Table 2), again highlighting female-to-male *chasing* and *attacking* as the most sensitive behaviors in this analysis. See also S3 and S4 Figs for supplementary details on behavioral bouts, and S5 Fig for age distribution across sexes and dyad types.

Therefore, dyad matching preferentially modulated female-to-male aggression in the undivided cage. Below, we link Experiments 1 and 2 via correlations within dyads.

### Linking divided cohabitation to undivided cohabitation: dyad matching affected correlations among behaviors

**Fig 4:**
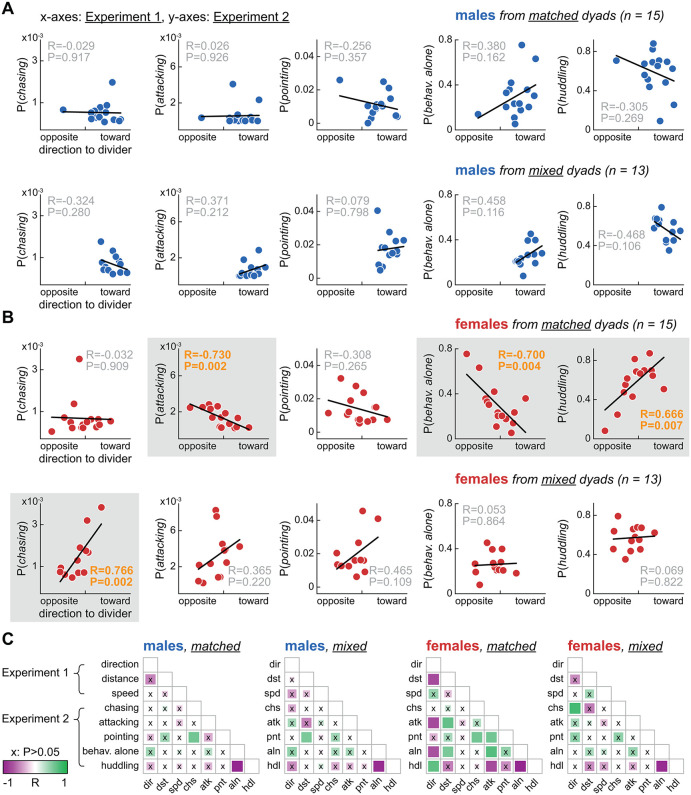
Linking divided cohabitation to undivided cohabitation: dyad matching affected correlations among behaviors. **A.** Correlations between the most sensitive variable from Experiment 1 (72-h divided cohabitation), i.e., direction to divider (x-axes), and behavior probabilities from Experiment 2 (4-h undivided cohabitation; y-axes), averaged per male individual (data points). **B.** Same but for females. Significant correlations revealed further distinctions between dyad types, highlighted by gray backgrounds. Matched-dyad females were more affiliation-driven: a higher tendency to behave toward the divider in Experiment 1 correlated with higher social affinity 3–4 days later, in Experiment 2 (lower likelihood to attack and behave alone, higher likelihood to huddle). This pattern was absent in mixed-dyad females; instead, they exhibited a positive correlation between direction to divider and *chasing* behavior. **C.** Comprehensive view of all correlation pairs. *R* and *P* values are coded by color and square size, respectively. Note that the scatter plots in panels A–B correspond to the leftmost column of each correlation matrix. See also S6 and S7 Figs. Underlying processed data and plotting code for this figure are available at figshare (https://doi.org/10.6084/m9.figshare.31820266).

The same dyad assignments were used in Experiment 1 (first male-female encounter and 72-h divided cohabitation) and Experiment 2 (second encounter and 4-h undivided cohabitation), with an interval of 3–4 days between experiments. This gave us the opportunity to quantitatively link these experiments. Thus, we averaged all behavioral variables per animal (three variables from Experiment 1, five from Experiment 2) and explored linear correlations among such variables. Fig 4A and 4B illustrates correlations between direction to divider in Experiment 1 and all behavioral categories of Experiment 2. Each data point in Fig 4A and 4B is an individual animal. Separate analyses were performed per sex and major dyad type (see Methods). The layout of Fig 4A and 4B includes significant correlations (gray background) as well as nonsignificant correlations, for visual contrast across sexes and dyad types.

Significant correlations were restricted to female voles and differed between matched versus mixed dyads (Fig 4B). *Chasing*: positively correlated with direction to divider in mixed. *Attacking*: negatively correlated with direction to divider in matched. *Behaving alone*: negatively correlated with direction to divider in matched. *Huddling*: positively correlated with direction to divider in matched (Fig 4B). Thus, a female that oriented more toward the divider in Experiment 1 was more likely to engage in different behaviors in Experiment 2 depending on the dyad type: affiliative behaviors in matched, chasing behaviors in mixed. This suggests that, depending on the dyad type, the same behavior in Experiment 1 (body orientation) may have been driven by a different underlying state. Fig 4C presents correlation matrices for all variable pairs, providing a comprehensive view of both sex and dyad type differences. The leftmost columns in each matrix of Fig 4C (direction to divider) contain the correlations emphasized above. Other correlations were found within each experiment, including negative correlations between *behaving alone* and *huddling*, consistent with their opposing trends in Experiment 2 (Fig 3). See also S7 Fig for scatter plots representing all significant correlations.

### Female-to-male behavior transitions differed between matched and mixed dyads

**Fig 5:**
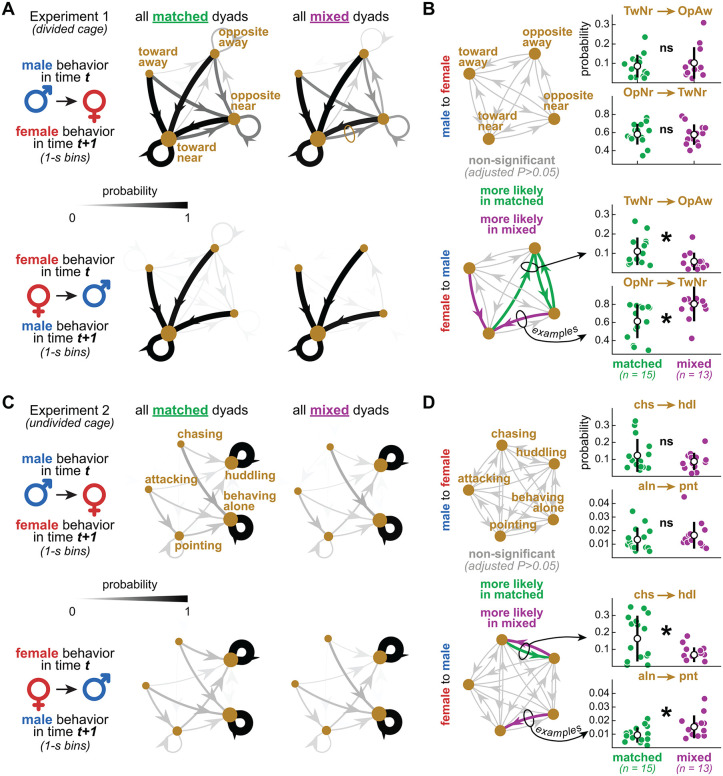
Female-to-male behavior transitions differed between matched and mixed dyads. **A.** Data from Experiment 1 (divided cohabitation). Direction and distance to divider were time-binned (1-s), categorized (toward-near, opposite-near, toward-away, opposite-away), and analyzed as male-to-female and female-to-male transition probability matrices (see Methods). Directed graphs (averaged across dyads) illustrate patterns that varied with both dyad type and sex directionality. **B.** Quantitative comparisons between dyad types within each sex directionality, using one transition matrix per dyad, rather than the averages in panel **A.** Behavior pairs were used as repeated measures in this analysis (see Methods). Self-loops were excluded to focus on inter-behavior relationships. The directed graphs highlight which transitions differed statistically between dyad types. The scatter plots exemplify the distributions underlying each transition edge (each data point is a dyad). Mean ± standard deviation bars accompany the scatter plots. C and D. Same as panels A and B but from Experiment 2 (undivided cohabitation). Overall, these patterns suggest that matched dyads showed more variation in body re-orientation behaviors between the females and males, as well as a higher likelihood of female *chasing* leading to *huddling*, among other interpretations. See Results and Discussion. See also Table 3 for statistics and S8 Fig. Underlying processed data and plotting code for this figure are available at figshare (https://doi.org/10.6084/m9.figshare.31820266).

The analyses above ignore within-dyad actions and reactions at finer temporal scales. How likely is behavior B in vole 2 after behavior A in vole 1? Do these likelihoods change with dyad type and/or sex directionality (male-to-female, female-to-male)? In Fig 5, we explore these questions using behavior transition probabilities, thus capturing temporal sequence patterns from each dyad type and sex directionality. Data from the video frames were divided into 1-s time bins; this temporal resolution was deemed optimal to capture all transitions between behaviors, including to and from aggression bouts—the quickest behaviors in our dataset (S2 Movie). We then conducted this analysis separately per major dyad type (matched, mixed) and sex directionality (male-to-female, female-to-male).

**Table 3:** **Statistics for Fig 5**.

Figurepanel	Sex direction	Effect	Value	
5B	Male-to-female	Dyad type	deg fr	1, 12
			F; *P*	=0.02; =0.904
		Behavior pair	deg fr	11, 132
			F; *P*	=123.86; <0.001
		Interaction	deg fr	11, 132
			F; *P*	=0.69; =0.747
	Female-to-male	Dyad type	deg fr	1, 12
			F; *P*	=0.01; =0.955
		Behavior pair	deg fr	11, 132
			F; *P*	=138.55; <0.001
		Interaction	deg fr	11, 132
			F; *P*	**=5.23; <0.001**
5D	Male-to-female	Dyad type	deg fr	1, 12
			F; *P*	=0.80; =0.389
		Behavior pair	deg fr	19, 228
			F; *P*	=70.03; <0.001
		Interaction	deg fr	19, 228
			F; *P*	=0.87; =0.618
	Female-to-male	Dyad type	deg fr	1, 12
			F; *P*	=5.13; =0.043
		Behavior pair	deg fr	19, 228
			F; *P*	=68.26; <0.001
		Interaction	deg fr	19, 228
			F; *P*	**=2.64; <0.001**

Degrees of freedom, F, and *P* values were obtained using two-way ANOVA with behavior pairs as repeated measures. *P* values < 0.01 were highlighted with bold font, except for repeated measure effects.

Fig 5A and 5B shows data from Experiment 1 (divided cohabitation). The most informative variables in that dataset—direction and distance to divider—were continuous and therefore required discretization prior to transition analysis. Thus, we discretized direction to divider into “toward” or “opposite”, and distance to divider into “near” or “away”, resulting in four categories: *toward-near*, *opposite-near*, *toward-away*, *opposite-away* (see Methods). The directed graphs in Fig 5A illustrate transitions among these categories, averaged across dyads. The edges represent probabilities, coded by line width and grayscale (Fig 5A). For this analysis, we focused on the initial 12 h of the 72-h recording, when the animals were more active (Fig 2).

The main visual pattern in all graphs is the convergence to, and recurrence of, *toward-near* (black edges): these represent the highest transition probabilities in both sex directionalities (Fig 5A). Particularly in the male-to-female graphs, the convergence to, and recurrence of, *opposite-near* (dark gray edges) also showed relatively high probabilities. All other transitions were less likely (light gray edges) (Fig 5A). The more distributed patterns in the male-to-female graphs indicate higher variation: female voles were more active and rotated themselves more (Fig 2 and S1 Movie), thus increasing the chances of male behaviors being followed by both body orientation categories in the females. In contrast, the female-to-male graphs indicate lower variation: all female behaviors were more likely followed by *toward-near* in the males, simply because this behavior was predominant in males (S1 Movie).

Of interest to this study, we found differences between matched and mixed dyads, specifically in female-to-male transitions (effect of interaction, F_11,132_ = 5.23, *P* < 0.001). For quantification, transition probability matrices were calculated per dyad, reshaped into dyad x edge matrices (edges were used as repeated measures), and compared between matched and mixed dyads, resulting in Fig 5B (self-loops were excluded; see Methods). In mixed dyads, two female-to-male transitions were more likely, as indicated by post-hoc effects: *opposite-near* to *toward-near* and *toward-away* to *toward-near* (Fig 5B). We interpret that this was driven by the tendency of mixed-dyad males to spend more time toward the divider, as reported earlier (Fig 2). In matched dyads, three female-to-male transitions were more likely: *toward-near* to *opposite-away*, *opposite-away* to *opposite-near*, and vice-versa for the latter (Fig 5B). These re-orientation patterns between male and female were infrequent (see light gray edges in Fig 5A) but could be speculated as body posture signals. Fig 5B additionally contains examples of the distributions behind each behavior pair, shown here as mean ± standard deviations (each data point represents a dyad). See Table 3 for statistics and S8 Fig for schematic illustrations of behavior transitions.

Fig 5C and 5D shows the same analysis, but from the full 4 h of Experiment 2 (undivided cohabitation), with five labeled behaviors during free interaction. As illustrated by the average directed graphs in Fig 5C, the highest transition probabilities were found in the recurrence of *behaving alone* and *huddling* (black self-loops). This was expected, as *behaving alone* and *huddling* were both prevalent and self-sustaining, as shown previously (Fig 3). For quantification, we excluded such loops to focus on the behavior pairs, as described above, resulting in Fig 5D. We again found differences between matched and mixed dyads, specifically in female-to-male transitions (effect of interaction, F_19,228_ = 2.64, *P* < 0.001). In mixed dyads, two female-to-male transitions were more likely, as indicated by post-hoc effects: *huddling-to-chasing* to *alone-to-pointing* (Fig 5D). This could reflect higher activity overall in mixed dyads, including shorter and more intermittent periods of *huddling* and more frequent *alone-to-pointing* re-encounters. However, these transitions showed very low probabilities (hardly visible in Fig 5C; see also y-axes in Fig 5D) and thus we interpret them cautiously. A more robust observation was that female-to-male *chasing-to-huddling* was more likely in matched dyads (Fig 5D). Thus, female *chasing* in matched dyads was possibly more affiliative, or huddling-seeking (S2 Movie). This suggests the existence of different states underlying the same behaviors depending on dyad type, echoing an interpretation we proposed in the previous subsection. See Table 3 for statistics and S8 Fig for schematic illustrations of behavior transitions.

Therefore, aside from the specificities of each behavior pair, Fig 5 shows that dyad matching influences actions and reactions on the scale of seconds, reinforcing the time series and correlation analyses reported earlier.

## Discussion

**Fig 6:**
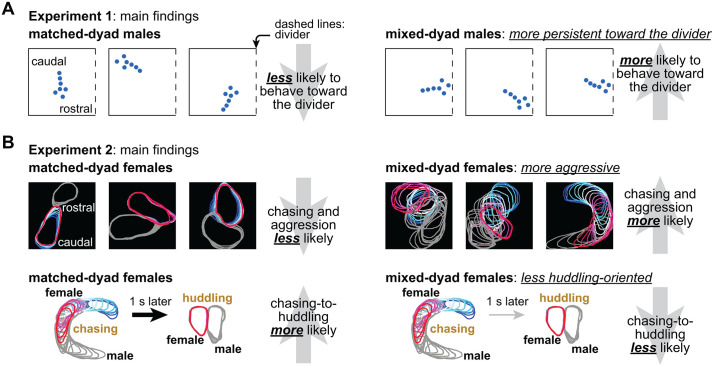
Summary of main findings. A. The main finding from Experiment 1 is that males from mixed dyads tended to behave more persistently toward the divider compared to males from matched dyads (Fig 2). No such effects were observed in females. B. The main finding from Experiment 2 is that females from mixed dyads were more likely to chase and attack compared to females from matched dyads (Fig 3). Correlations between Experiments 1 and 2 reinforced the influence of dyad type on social behaviors (not illustrated here; see Fig 4). Furthermore, matched-dyad females were more likely to transition from *chasing* to *huddling* (Fig 5), even though *chasing* was less frequent in females from matched dyads (Fig 3).

We utilized algorithms for annotation and decomposition of social behaviors to examine dyad matching in rodents. Opposite-sex prairie voles were assigned to either matched or mixed-dyad types, resembling human neurotype matching studies. The voles were then recorded in two experiments: initial divided and subsequent undivided cohabitation. Dyad type effects emerged from both experiments in a correlated manner, suggesting robust underlying mechanisms. Our observations varied by sex and spanned multiple temporal scales, including body orientation patterns over days, incidence of aggression over hours, and behavioral transitions over seconds. These findings might influence further development of behavioral paradigms and studies integrating social behavior and physiology. See Fig 6 for a graphical summary of our main findings.

### Juvenile sleep and later-life social behavior

Neurotype matching studies in humans have quantified behavioral and physiological markers of rapport by assigning socially different individuals into matched or mixed dyads [4–10,17,19,21–23]. Here, “social difference” was generated in adult prairie voles by pre-exposing them to either control conditions or ELSD during post-natal days 14–21—a method known to modulate adult social behavior, as described by our group [35,41].

For context, ELSD causes fragmentation of NREM and reduces both duration and gamma power of REM, as recorded electrographically from prairie vole pups. Body weight, corticosterone levels, and parental care are all unaffected by ELSD, indicating that this method selectively targets juvenile sleep physiology [35,41]. Despite this selectivity, ELSD provokes long-lasting behavioral and neurobiological alterations into adulthood. Behavioral alterations include reduced male-to-female partner preference expression, as assessed via the three-chamber partner preference test (PPT) [35]. In the PPT assay, a freely-moving vole interacts with either of two tethered voles, one familiar and one stranger. Our previous work [35] showed that ELSD causes male voles to huddle less with familiar females in the PPT assay, indicating diminished partner preference. In the present study, we show that ELSD additionally affects dyadic behaviors in two home-cage setups (divided and undivided), revealing novel social dynamics that are less evident in the PPT assay, including body orientation and social role changes. In terms of neurobiology, ELSD has been shown to affect cytoarchitectural markers of excitatory-inhibitory imbalance in brain regions involved in sensory and cognitive processing, such as increased expression of inhibitory neurons in somatosensory cortex, as well as increased dendritic spine density and decreased presynaptic glutamate labeling in prefrontal cortex [35,42]. Therefore, prairie vole ELSD continues to reveal distinct outcomes across behavioral and neurobiological assays, reinforcing its value for investigating the myriad of sequelae linked to poor juvenile sleep [43].

As reviewed previously [43–45], sleep disruption during infancy has been particularly associated with ASD in humans. Brain maturation processes in humans, such as axonal myelination and synaptic pruning, depend on healthy sleep during development. Disrupted sleep may interfere with these processes and increase vulnerability to ASD-like symptoms, including social difficulties [43–45]. Since dyad matching modulates behavior in both prairie vole ELSD (present study) and human ASD [4–8,17,19,21–23], we cautiously speculate that at least some of these social properties may trace back to sleep quality during development. This is, of course, a much more complex question. First, the relationship between juvenile sleep disruption and ASD in humans is recognized as a bidirectional and multifaceted comorbidity, with sleep disturbances both amplifying and being amplified by ASD [44,45]. This bidirectionality has also been demonstrated in a mouse study co-examining ELSD and genetic risk, with homozygous Shank3 mice (an ASD model) intrinsically sleeping less, and heterozygous Shank3 mice (particularly males) being more sensitive to the ELSD effects on social behavior than wild-type controls [46]. Second, in addition to genetic factors, human ASD is linked with environmental factors not directly related to sleep, such as perinatal exposure to toxicants [47]. And third, juvenile sleep is sensitive to circumstances not directly related to ASD, such as respiratory problems and household conditions [48,49]. All these factors can lead to (and interact with) ASD [50], making it difficult to isolate the role of juvenile sleep in ASD, let alone dyad type properties. Still, our results suggest that juvenile sleep does play a role in these social properties—a role that we found to be discernible in nonhuman animals, despite the elusive mechanisms. Future studies could disentangle poor juvenile sleep from underlying factors (e.g., Shank3 zygosity [46]), furthering the study of nonhuman dyad matching and complementing human research.

### Markers of affinity across social phenotypes

As contextualized above, most dyad matching studies in humans have focused on ASD. Human ASD encompasses a myriad of features, including subtle presentations that can be challenging to detect and often go undiagnosed [51,52]. Likewise, ELSD prairie voles do not show overt behavioral changes. In our experience, these changes can only be detected with quantification, whether using manual [35] or computerized [38] tracking. Thus, we cautiously speculate that ELSD prairie voles might represent a nuanced social difference phenotype, analogous to nuanced forms of ASD in humans. Our experiments add complexity to this question. Despite the elusive nature of these phenotypes, they can still supply us with controllable “reagents” to manipulate “social chemistry”. The “purity” of these reagents (i.e., phenotypes) certainly vary among species or between laboratory and naturalistic settings, yet they still seem to bring about some form of chemistry, as suggested by the literature on human dyads and our study in prairie voles. Furthermore, dyadic behavior in humans has been investigated beyond ASD, including studies on dysphoria, attention deficit disorder, and decision-making [3,11–13], all of which reinforcing that mixing or matching different phenotypes (or social styles) can modulate cooperative and social behaviors in quantifiable manners. This indicates that social matching properties are measurable across species and phenotypes, with implications for basic science, mental health, and a better understanding of reciprocity and empathy [25–27].

These questions warrant further investigation using data-intensive metrics of social affinity. Recent human ASD studies involving matched and mixed dyads used objective metrics from video, audio, and electroencephalography, including motion energy, mutual gaze, backchanneling, verbosity, smile synchrony, and inter-brain synchrony [16,17,19,21,22]. Generally, these studies also support neurotype matching properties, though some metrics are inconsistent with this trend. Such inconsistency might be attributed to low temporal resolution, as these studies used aggregated metrics across sessions when comparing dyad types. Time series analyses, like those here, could be employed in future studies to extract detailed information from each metric, potentially identifying optimal metrics for real-time social behavior tracking and translatability across species. Other human dyad studies, unrelated to ASD or neurotype matching, used additional metrics, including task cooperation, motor coordination, smell-induced arousal, and functional brain imaging or spectroscopy [2,18,24,53–57]. This variety of methods suggests promising avenues for studying dyadic behavior across species and neuropsychiatric disorders.

### Interaction between biological sex and social differences in controlled settings

In our male-female grouping design (Ctrl-Ctrl, ELSD-ELSD, Ctrl-ELSD, and ELSD-Ctrl), we considered both sex and dyad type. This revealed dyad type effects both between and within sexes in prairie voles. Interactions between and within biological sexes have also been recognized in human ASD. Core ASD traits seem to vary equivalently between biological sexes (e.g., communication impairments, repetitive behaviors), whereas associated ASD traits seem to vary independently within each sex (e.g., hyperactivity in males, depression in females) [58–60]. Sex and social difference interactions have also been examined in the ASD-relevant mouse study cited above, i.e., the study on the behavioral effects of environmental (ELSD) and genetic factors (Shank3 heterozygosity) [46]. According to this study, the combination of ELSD and Shank3 heterozygosity reduces male (but not female) responsivity to social stimuli, and increases female (but not male) tolerance to a nonsocial anxiogenic environment (elevated plus maze) [46]. Thus, male and female mice exposed to (and/or intrinsically carrying) ASD-like risk exhibit different behavioral profiles across social and nonsocial paradigms. This interplay between sex and social differences in rodents speaks to the heterogeneity of ASD presentations in humans, which can be further confounded by cultural factors (e.g., camouflaging or “masking” of social difficulties by biological females [61–63]), and insufficient diagnostic criteria (biased toward biological males) [46,58–60]. These are all complex questions: gaps remain in our understanding of both biological and cultural contributions to social differences.

These knowledge gaps become even more apparent if we consider the supra-individual properties that can arise from dyad matching experiments, including (but not limited to) those relevant to ASD. As shown here with prairie voles, sex differences in body direction (males spent more time toward the divider) and aggression (females chased and attacked more) were exacerbated in mixed dyads. We can now hypothesize that dyad matching could also amplify sex differences across species, broadening the scope of social difference research beyond focal-animal analyses, like partner preference or short-term social stimulation [35,46]. However, these insights remain largely speculative, as our male-female dyad design was influenced by its inherent reproductive context. This aspect limits the comparability of our results to the available dyad matching literature, which has so far focused on nonreproductive dyadic interactions in humans, such as task cooperation or perceived friendship potential [4–10,17,19,21–23]. Addressing this limitation will require more research. Specifically, additional rodent and human studies are needed to quantify dyad matching more systematically, especially in the context of opposite-sex interactions.

### Ecological perspectives on biological sex and social differences

The speculations above are more relevant to the psychological and clinical aspects of social compatibility, which are amenable to controlled experimentation both in humans and other species. However, broader ecological implications are also applicable. Quantifiable social matching may well be occurring “out there”, both in the wild and in human groups, driven by natural individual variations. Can we quantify these phenomena more systemically in different dyad types (opposite-sex, same-sex) and larger social groups with higher combinatorial complexity? Emerging socioecology research that accounts for individual variations increasingly support that direction [64–68].

Sex-specific dynamics are worth emphasizing in this ecological scenario. In the wild, mate choice is a complex phenomenon, mostly dictated by females [69]. Among various mating strategies, social monogamy is found in roughly nine percent of mammalian species [70], and is thought to have evolved primarily as a means to enhance breeding success, rather than solely enabling paternal care—a notion to which prairie vole research has contributed [71,72]. In turn, female mate choice is often influenced by male courtship displays, which in prairie voles include enhanced rates of anogenital sniffing [73,74]: a behavior also observed in our study and further discussed below. In reproductive contexts like our experiment, aggressive reactions of the female-to-male sniffing attempts can predict diminished sexual receptivity [75], akin to the importance of early-phase affinity to long-term relationships in humans [1]. Therefore, early-phase female receptivity and male courtship may predict the strength of the eventual pair bond.

With this ecological framework in mind, we can now reflect on behavioral nuances revealed by our two experiments. We found that female body orientation in divided cohabitation (Experiment 1) led to different outcomes in undivided cohabitation (Experiment 2) depending on the dyad type: increased affiliation in matched dyads (less attacking, more huddling) and decreased affiliation in mixed dyads (more chasing, less *chasing-to-huddling* transitions; Fig 6). Therefore, a female behavior that looked the same in Experiment 1—body direction—may have been driven by different underlying states depending on the dyad type, e.g., female body direction was possibly more huddling-oriented in matched dyads. Interestingly, these states in females may have influenced (and been influenced by) the males. In mixed dyads, males spent more time toward the divider in Experiment 1, and once the divider was removed in Experiment 2 the females attacked more frequently (Fig 6). Thus, females may have reacted proportionally to the males’ approaches upon removal of the divider, which could be another explanation why aggression was more frequent in mixed dyads. Therefore, we speculate that dyad type, as manipulated here, may have revealed a dissociation between expressed behaviors and underlying states, with female body direction being more driven by aggression or affiliation states depending on dyad type. Dyad type may have similar influence on other internal states, including aggression and courting, but also foraging, social hierarchy, familiarity, and biological sex of the conspecific [76–78]. We propose that matching and mixing rodent “neurotypes” like we did here is a promising approach to investigate these internal states, with implications for socioecology studies like the ones cited above.

### Concluding remarks

This study suggests that social compatibility is a quantifiable phenomenon in rodents. As computational ethology continues to optimize social behavior tracking, the emergent properties of social groups may become more tangible. This progress may reshape behavioral paradigms, from dyad-based studies to freely-moving partner preference studies, strengthening the link between animal models and human social behavior.

## Materials and methods

### Subjects

Prairie voles (*n* = 28 each sex) were sourced from a genetically diverse pool of 13 breeders in an accredited, fully-contained laboratory facility (VA Portland Health Care System). Litters containing both male and female sibling pups (2–9 pups each breeder pair, 13 unique breeders) were raised with their parents (no cross fostering) until post-natal day 21 (P21). During the P14–21 period, specifically, the litters were subjected to either control (Ctrl) or ELSD conditions (details below). At P21, pups were weaned into same-sex sibling groups and remained at the same vivarium until approximately P90. The sibling groups were then shipped to the University of Michigan Medical School for the primary recordings of this study.

Consistent housing conditions were maintained across both institutions, including 14:10 h light/dark cycle (lights on at 5:00 am), temperature (20–23 °C), ventilation (double-filtered outside air, negative pressure), humidity (30%–70%), bedding (rolled paper pellets), environmental enrichment (cotton nestlets and wooden blocks/sticks) and *ad libitum* water and food (rabbit chow, corn, cracked oats). Each week, the animals were transferred to a new cage with fresh bedding and enrichment. All procedures were carried out in accordance with the National Institutes of Health Guide for the Care and Use of Laboratory Animals, and approved by the Institutional Animal Care and Use Committees (IACUC) of VA Portland Health Care System (protocols 6069 and 3652) and University of Michigan Medical School (protocol 00011423).

Our animals originated from a colony at Emory University, itself derived from wild-captured animals in the State of Illinois, USA. To preserve genetic diversity, bi-annual exchanges of animals occur among several institutions, including North Carolina State University, University of California Davis, University of Colorado Boulder, and Florida State University.

### Early-life sleep disruption

Prairie vole ELSD phenotypes of both sexes were generated by placing home cages with P14–21 pups and their parents on an orbital shaker for intermittent gentle agitation (10 s on, 100 s off, 110 rotations per minute). ELSD animals were provided hydrogel instead of water bottles to prevent water spillage during agitation. Ctrl animals experienced the same conditions except for agitation (Fig 1A). As previously described by our group [35,41,42], ELSD disturbs sleep in prairie vole pups without affecting their hormonal markers of stress, or the parental care they receive. The acute effects of ELSD are, therefore, confined to sleep electroencephalography (EEG) parameters, including fragmentation of NREM sleep and reduction of REM sleep time by more than 20% over a 24-h period [35]. Given this support from our previous studies, here we used identical cage agitation parameters without EEG validation, resulting in a noninvasive (purely behavioral) experimentation timeline.

### Dyad assignment and age at recording

Following the ELSD or Ctrl procedures at P21, the pups were weaned into same-sex groups of 2–4 siblings per cage until adulthood, resulting in four types of sibling groups (Male-Ctrl, Male-ELSD, Female-Ctrl, and Female-ELSD). As mentioned above, animals were sourced from a genetically diverse pool of 13 unique breeders, each of whom contributed a single litter containing both male and female pups (2–9 pups each litter). Sexually naïve opposite-sex adults were then randomly assigned into matched dyads [Male-Ctrl/Female-Ctrl (*n* = 7), Male-ELSD/Female-ELSD (*n* = 8)] or mixed dyads [Male-Ctrl/Female-ELSD (*n* = 7), Male-ELSD/Female-Ctrl (*n* = 6)] prior to recordings, for a total of 28 dyads (Fig 1A).

Our recording setup could accommodate up to four dyads at a time (details below). Since there were 28 dyads in total, the recordings were divided into seven cohorts of four dyads. Furthermore, an interval of 2−3 weeks was necessary between cohorts due to the duration of the experiments combined with preparatory activities, like cage cleaning and file management. This resulted in a 4-month period to record all 28 dyads. Thus, the ages of the animals at the time of recording varied with a mean ± standard error of 185.8 ± 6.5 days of age. However, the individuals of each dyad were still age-matched, with an age difference of 11.1 ± 2.2 days, randomly distributed across dyad types. See also S5 Fig for more details on age distributions across sexes and dyad types.

### Recording setup

For recording, we developed a vibration-free optomechanical assembly using metal parts from a vendor (ThorLabs), as described previously [38]. This setup holds two infrared-sensitive grayscale cameras (Basler, acA1300-60 gm) positioned at 90° overhead angle. Each camera was mounted to a fixed focal length lens (Edmund Optics, 6 mm UC Series) and surrounded by four infrared LED illuminators. Each camera/lens was able to frame two home cages, hence the ability to record four dyads at a time. This system was installed in a room with light/dark phases, which however did not affect imaging brightness, as it relied on infrared reflectance. The cameras were connected to a computer via a network adapter (Intel Pro 1000/PT), and data were stored on a redundant array of independent disks (RAID). Recording settings were configured via Pylon software (Basler) with the following parameters: 8-bit grayscale depth, frame width x height of 800 × 896 pixels, no binning, 1,328 kbps data rate, 20 Hz frame rate. Exposure and brightness were adjusted prior to each recording by manipulating the lenses and infrared illuminators, without digital adjustments. Data were acquired into mp4 files (H.264 coded) using StreamPix software (NorPix). For the 72-h recordings of Experiment 1, specifically, recordings were acquired into 6-h mp4 files to ensure file readability, including 2-s margins at the beginning and end of each 6-h file.

### Experiments 1 and 2

Experiment 1 consisted of divided cohabitation for 72 h starting at 8 am, i.e., 3 h after the onset of the light phase. Each male-female pair was placed in a bedded home cage (48.3 cm length, 25.4 cm width, and 20.3 cm height) with a lab-made divider at the center of the cage. Thus, each animal was spatially-restricted to a quadrant, producing uniform behavioral variables across recordings (Fig 1B). The dividers were made of metal wire-mesh (square mesh, 6.5 mm aperture, 22 cm wide, and 28 cm high) covered on both sides with clear polycarbonate sheet (0.5 mm thick) to prevent animals from climbing. The bottom section of the metal mesh, measuring 6.5 cm in height, was left exposed without the polycarbonate sheet, allowing animals to exchange bedding, smell each other and engage in face-to-face, and body-to-body interactions. Chow and gel were placed at consistent locations away from the divider, on the left and right sides of the animal, respectively. No environmental enrichment objects were provided, encouraging the animals to interact. A handwritten label was placed outside each quadrant displaying animal metadata (sex, ELSD versus Ctrl, and quadrant number), clearly visible in each recording for extra documentation. Cardboard barriers were placed between adjacent cages, preventing different dyads from seeing each other during the multi-dyad recordings.

Experiment 2 consisted of undivided cohabitation for 4 h, also beginning at 8 am, 3–4 days after the end of Experiment 1; animals were single-housed at the vivarium between experiments. Before each recording of Experiment 2, the male and female were placed in the same home-cage quadrants, with the same handwritten label, same bedding and without chow or gel. We then removed the divider *while recording*, ensuring animal traceability throughout the recording, as male and female lacked physical differentiators (Fig 1C). See the next section for further identity verification methods. The male and female were then allowed to interact freely. At the end of the recording, each animal was visually inspected to confirm sex, resulting in a 100% match with the original metadata.

### Data pre-processing and animal tracking

For Experiment 1, each camera recorded two divided cages and, therefore, four quadrants, one animal per quadrant. Thus, we decided to crop each video into four smaller videos (384 × 416 pixels), one per quadrant, using Adobe Premiere and Media Encoder. By doing this we eliminated the need for identity verification in Experiment 1 (unlike Experiment 2, as described below). The cropped videos were then processed through DeepLabCut, a supervised keypoint tracker [33]. A resnet-50 network was used to label seven keypoints per animal: nose, left/right ears, three locations along the “spine”, and tail base (Fig 1B and S1 Movie). The network was trained with video frames from three males and three females, each frame representing varying levels of difficulty: from clear imaging of the animal (without obstruction of body parts) to challenging situations (e.g., curled posture when sleeping or eating, tail base hidden under bedding, nose hidden by the mesh divider). The network was trained using a laboratory server (CPU: Intel Xeon E5-2640 v3 @ 2.60 GHz. RAM memory: 512 GB. GPU: NVIDIA GeForce GTX 1080 Ti) and deployed to unseen (held-out) data. Once keypoint coordinates were generated for each recording, we proceeded to behavioral decomposition analysis using MATLAB (MathWorks), as detailed in the next section.

For Experiment 2, all data processing and behavior annotation steps were performed using LabGym2, an open-source machine vison tool that we co-developed (including authors: Y.H., B.Y., and L.S.B-J.) [39]. First, each raw recording was cropped into two rectangular framings (800 × 450 pixels), one per cage, so that each video contained one male-female dyad. The cropped recordings were then contrast-enhanced to highlight the animals against the background (bedded cage). These were then analyzed using two neural networks that we trained during our previous study: *Detector* and *Categorizer* [39]. Step 1: Segmentation and masking of each animal using the *Detector*. Step 2: Mask-based foreground extraction to obtain the raw pixels of each animal; background pixels were set to zero (black). Step 3: Unsupervised tracking of masks over consecutive frames based on foreground appearance and distance. Step 4: Generation of spatiotemporal patterns as inputs for the *Categorizer*, including body contour outlines around the animals. Specifically for the focal-animal, these outlines were color-coded from blue to red to indicate the temporal progression of movements within groups of frames, i.e., time bins totaling 0.67 s per bin. The nonfocal (or reference) animal was color-coded in a fixed tone of gray (Fig 1C). The focal and reference roles were alternated per animal by processing each recording twice, enabling social role identification [39]. Time bin size (0.67 s) was determined to match the timescale of prairie vole behavior, including quick bouts of chasing and aggression. Step 5: Categorization of spatiotemporal patterns into behaviors (Fig 1C). This step involved manual sorting of behavioral examples—the primary supervised aspect of LabGym2—followed by deep learning of the spatiotemporal features of these behaviors using a custom neural network structure, as detailed in our software publication [39]. LabGym2 was run remotely on the University of Michigan Great Lakes cluster. The sessions on this cluster were configured with the following resources: 2.9 GHz Intel Xeon Gold 6226R with 372 GB RAM, NVIDIA A40 with 48 GB VRAM.

LabGym2’s publication included a proof-of-concept study for prairie vole dyads, demonstrating 100% accurate individual animal tracking throughout each recording [39]. However, the recordings in that study were relatively short (10 min), whereas the recordings in the present study were longer (4 h), which we found to increase susceptibility to identity switching. Tracking identities over extended periods (e.g., several hours) indeed remains a challenging task in the field of computational ethology, despite the development of various approaches to multi-animal tracking [31–34]. In our experience, species-typical *huddling* behaviors exacerbated the risk of identity switching, especially 2–3 h into the recording, when the voles tended to huddle more. Thus, we chose to identify male and female *manually*, independently on LabGym2’s behavioral categorization. Additionally, we leveraged this manual inspection effort to annotate “*attacking*” and “*being attacked*” events (S2 Movie), which we could not reliably automate in the current dataset. The annotation of “*chasing*” and “*being chased*”, on the other hand, was fully automated, serving as a control for our findings on sex-specific aggression roles. For the manual annotation of animal identities and aggression roles we used the graphical user interface (GUI) of another software tool: DeepEthogram [79].

In summary, we employed two distinct machine vision pipelines in this study, each tailored to a recording environment (divided and undivided cohabitation). However, our study also involved significant human supervision, including manual validation of sex identities and aggression roles, mitigating the risk of over-automation [80]. In fact, our results on “*chasing*” and “*attacking*” supported each other (Fig 3), which is an important control, as “*chasing*” and “*attacking*” were annotated by computer and human, respectively. We believe that human-in-the-loop strategies like these will continue to offer a balance between effort cost and ethological validity, revealing meaningful patterns like the dyad matching effects reported here.

### Behavioral decomposition and categorization

Experiment 1: Keypoints from DeepLabCut represented each animal as an arrow-shaped “skeleton”, one per video frame (Fig 1B). The coordinates for these keypoints were imported into MATLAB, converted from pixels to centimeters, and processed into three behavioral variables.

1. Direction to divider: a line was fitted between the extreme keypoints (nose and tail base), and the angle of this line relative to the horizontal plane of the frame was calculated. This angle was then rescaled from −1 (opposite from the divider) to +1 (toward the divider), with clockwise and counterclockwise directions treated equally.
2. Distance to divider: horizontal distance between the center of the animal and the divider, regardless of its position along the vertical axis.
3. Locomotion speed: difference (hypotenuse) between the center of the animal in frame n and frame n + 1.

These measures were timestamped according to clock time, in number of seconds × number of frames. The clock was not restarted at midnight, assigning each frame to a unique temporal identifier across the 72 h. This allowed us to trim and align the recordings to a common 72 h axis, as safety margins of a few minutes were recorded before and after each session. Clock time information was obtained from file naming metadata through the acquisition software (StreamPix, NorPix). These procedures replicate those used in a previous publication [38]. See S1 Movie.

Experiment 2: LabGym2’s outputs include the likelihood of each behavioral category per video frame on a 0–1 scale. The categories were initially the same as our proof-of-concept study in prairie voles [39]: *aggression*, *chasing*, *being chased*, *approaching*, *sniffing*, *being sniffed*, *moving alone*, *idling alone*, and *huddling*. For the present study, aggression was ignored (replaced by manual annotations, as described below) and adaptations to other categories were made using MATLAB.

1. *Sniffing* into *Pointing*: this category merged three types of *sniffing* (face-to-face, face-to-body, and anogenital) and *approaching* behaviors. This was renamed to a generic term, “*pointing*”, as freezing-like behaviors that typically occurred after aggressive bouts (S2 Movie) were often misclassified as “*sniffing*” under LabGym2’s spatiotemporal patterning. Neural network refinement and deeper ethological exploration were outside our scope (e.g., sub-classification of *sniffing*, sequencing of peri-aggression postures).
2. *Behaving alone*: this category merged “*moving alone*” and “*idling alone*” (this latter included self-grooming). In-depth exploration of these solitary behaviors was outside our scope, hence the creation of this generic category. Frames were set to zero likelihood of “*behaving alone*” if only one animal was classified under this category, making it a full-dyad category (see purple curves in Fig 3).
3. *Huddling*: same as the previous proof-of-concept study in 10-min recordings [39], but with one correction that we found necessary in the present 4-h recordings. During intense body contact, the identities of both animals sometimes merged into one, causing “*huddling*” to be misclassified as “*idling alone*”. Frames with this issue we re-classified as *huddling*. Frames were set to zero likelihood of “*huddling*” if only one animal was classified under this category, making it a full-dyad category (see purple curves in Fig 3).

After these adaptations, likelihood values were converted into a vector of integer labels using a winner-takes-all approach. Finally, frames manually classified as “*attacking*” and “*being attacked*” were superimposed on that vector of labels. For final quantification (explained next), the focal roles of *chasing*, *attacking*, and *pointing* were analyzed without their mirroring counterparts (i.e., *being chased*, *being attacked*, *being pointed at*), thus minimizing redundancy in the figures.

### Final data processing and statistical analysis per figure

Figs 2 and 3: Time series data obtained from the behavioral decomposition (Experiment 1) and behavioral annotation (Experiment 2) methods above were analyzed, with each data point corresponding to one video frame of a single animal. These data points were binned and smoothed on a per animal basis: the 72-h recordings of Experiment 1 were averaged into 1-h bins (total of 72 bins) and the 4-h recordings of Experiment 2 were averaged into 3-min bins (total of 80 bins), both followed by a 6-bin moving mean window. The binned data were then plotted as ± standard error curves comparing males versus females overall, males versus females within major dyad groups (matched or mixed), males versus females within-dyad subgroups (Ctrl-Ctrl, ELSD-ELSD, Ctrl-ELSD, and ELSD-Ctrl), and matched versus mixed dyads within each sex. These comparisons were performed comprehensively across behavioral variables, allowing to identify sex or dyad type differences with both: (1) behavioral variable specificity (in which behavioral variables the dyad type precipitated or amplified differences?); and (2) temporal specificity (at which time bins did these differences emerge?). Statistical comparisons were made using two-way ANOVA with time bins as repeated measures, followed by Tukey’s post-hoc tests per time bin. The main statistical effects (sex, time, and interaction) are reported in Tables 1 and 2 and significant post-hoc differences are depicted as bold lines along the x-axes in Figs 2 and 3. Distributions per repeated measure passed the Kolmogorov-Smirnov normality test.

Fig 4: Experiments 1 and 2 were performed in different environments (divided and undivided cohabitation), 3–4 days apart. The animals and dyad assignments were the same between experiments, allowing for correlation analyses among all eight variables from the two experiments. To investigate this, behavioral variables were averaged across the entirety of each experiment, resulting in eight averages per animal. These averages were then grouped by sex and major dyad type (males or females from matched or mixed), forming four animal × variable arrays. Linear correlations within each sex and dyad type group were calculated, producing matrices with correlation coefficients (R) and corresponding *P* values. Key correlations were illustrated as scatter plots with linear fits in Fig 4A and 4B, while the *R* and *P* value matrices themselves were illustrated with a color code in Fig 4C. Distributions per variable passed the Kolmogorov–Smirnov normality test.

Fig 5A and 5B: Behavior transition probabilities were computed using 1-s time bins, matching the approximate timescale of prairie vole behaviors, including chasing and aggression bouts. For Experiment 1, continuous DeepLabCut-based variables of direction and distance to divider were averaged per time bin and discretized into categorical vectors (number of elements = number of time bins), enabling transition probability analysis. For discretization, direction to divider was converted from a −1 to 1 scale into “toward” versus “opposite”, whereas distance to divider was converted from a centimeter scale into “near” versus “away”. These categories were combined into a four-category vector (toward-near, opposite-near, toward-away, and opposite-away), one per animal. Then each dyad had a “time t” animal and a “time t+1” animal assignment, one iteration per sex directionality (male-then-female, female-then-male). Each of these iterations resulted in a 4 × 4 adjacency matrix (4 = number of behavioral categories), with rows representing “time t” behaviors, columns representing “time t+1” behaviors, and values indicating probabilities. Multiple such adjacency matrices were created, grouped by both sex directionality and dyad type. Specifically for the illustrations in Fig 5A, adjacency matrices were averaged across the dyads of each group and plotted as directed graphs. The edges of these graphs were coded by both weight and grayscale: the thicker and darker the edge, the higher the transition probability. For quantification (Fig 5B) no averaging was applied. Instead, each adjacency matrix (one per dyad) was reshaped into a row vector containing all behavior pairs (“behavior in time t” and “behavior in time t+1”). These rows were then concatenated across dyads, creating dyad × behavior pair matrices. Finally, dyad × behavior pair matrices from matched and mixed dyads were statistically compared per sex directionality using two-way ANOVA with behavior pairs as repeated measures, followed by Tukey’s post-hoc tests per behavior pair. The main statistical effects (dyad type, behavior pair, and interaction) are reported in Table 3 and significant post-hoc differences are depicted as bold edges in Fig 5B. Distributions per repeated measure passed the Kolmogorov–Smirnov normality test.

Fig 5C and 5D: Similar methods were used for Experiment 2 with three differences: (1) LabGym2-based data were already in categorical form prior to analysis, eliminating the need for categorization; (2) each time bin (1 s) was assigned its most frequent category (winner-takes-all); and (3) this analysis included five behavioral categories (*chasing*, *attacking*, *pointing*, *behaving alone*, and *huddling*), leading to 5 × 5 adjacency matrices.

## Supporting information

S1 Movie Decomposing behavioral variables in the divided cage. (MP4)

S2 Movie Examples of behaviors in the undivided cage. (MP4)

S1 Fig Behavioral bout structure from Experiment 1 variables—focus on sex differences within matched and mixed prairie vole dyads. (PDF)

S2 Fig Behavioral bout structure from Experiment 1 variables—focus on dyad type differences within prairie vole sexes. (PDF)

S3 Fig Behavioral bout structure from Experiment 2 variables—focus on sex differences within matched and mixed prairie vole dyads. (PDF)

S4 Fig Behavioral bout structure from Experiment 2 variables—focus on dyad type differences within prairie vole sexes. (PDF)

S5 Fig Examining prairie vole age as a covariate: no evidence that age confounded our dyad type comparisons. (PDF)

S6 Fig Mutual and/or sex-unspecific behavioral variables in prairie voles, using dyads (not individuals) as the units for statistical variation. (PDF)

S7 Fig Supplementary scatter plots for Fig 4. (PDF)

S8 Fig Illustration of male-to-female and female-to-male behavior transitions in 1 s bins—supplementing Fig 5. (PDF)

## Abbreviations

ASD: autism spectrum disorders
EEG: electroencephalography
ELSD: early-life sleep disruption
GUI: graphical user interface
IACUC: Institutional Animal Care and Use Committees
NREM: non-rapid eye movement
PPT: partner preference test
RAID: redundant array of independent disks
REM: rapid eye movement.
