# Supplementary material for: Social compatibility in opposite-sex prairie vole pairs is modulated by early-life sleep experience
Source: PLoS Biol. 2026 Mar 27;24(3):e3003434. doi: 10.1371/journal.pbio.3003434 (PMC13043049; doi:10.1371/journal.pbio.3003434)
Supplement: S1 Fig — (PDF) [file pbio.3003434.s003.pdf]

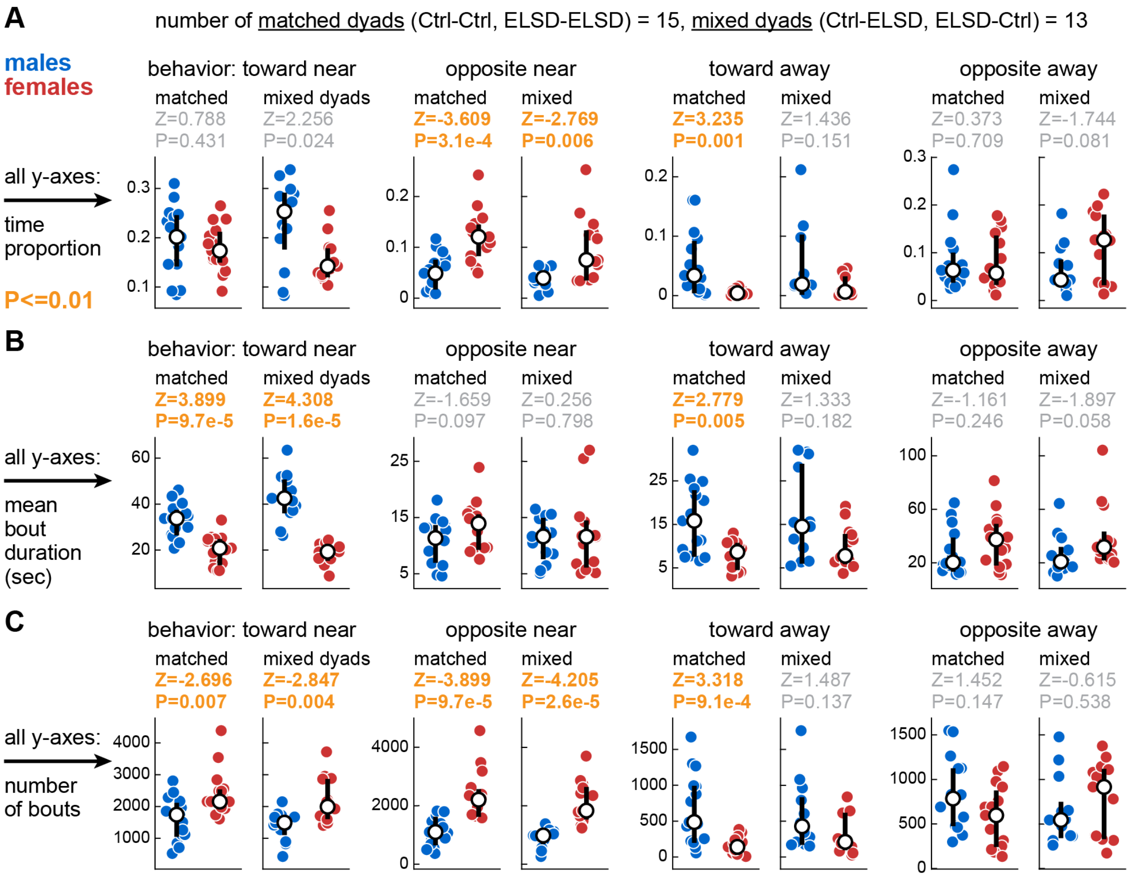

S1 Fig. Behavioral bout structure from Experiment 1 variables – focus on sex differences within matched and mixed prairie vole dyads. A. Time proportions (y-axes) for each behavior (four subplot columns) were calculated per individual animal (data points) and quantified for sex differences within dyad types using median and interquartile ranges (black circles/bars), alongside Wilcoxon rank sum tests (Z and P values). Significant effects are highlighted in orange font. The four behavior categories – "toward near", "opposite near", "toward away", "opposite away" – were defined by discretizing two continuous variables from Experiment 1: direction to divider and distance to divider, both derived from a keypoint tracker (DeepLabCut) as described in the main manuscript (see Methods and **Fig 5** description in the Results). **B-C.** Same layout, but showing mean bout duration and number of bouts on the y-axes. Main takeaway: the greater tendency of mixed-dyad males to behave "toward near" the divider is primarily explained by bout duration, more than the number of bouts, adding context to the time-series effects in **Fig 2B**. See **S2 Fig** for within-sex analyses, which corroborate bout duration as a key driver of "toward near" behaviors. Other sex differences, or lack thereof, in this figure collectively show varying effects of dyad type, suggesting that the ethology of rodent dyad matching is multifaceted and highly amenable to quantification. ELSD: early-life sleep disruption. Ctrl: control. Underlying processed data and plotting code for this figure are available at figshare (https://doi.org/10.6084/m9.figshare.31820266).
