# Supplementary material for: Social compatibility in opposite-sex prairie vole pairs is modulated by early-life sleep experience
Source: PLoS Biol. 2026 Mar 27;24(3):e3003434. doi: 10.1371/journal.pbio.3003434 (PMC13043049; doi:10.1371/journal.pbio.3003434)
Supplement: S2 Fig — (PDF) [file pbio.3003434.s004.pdf]

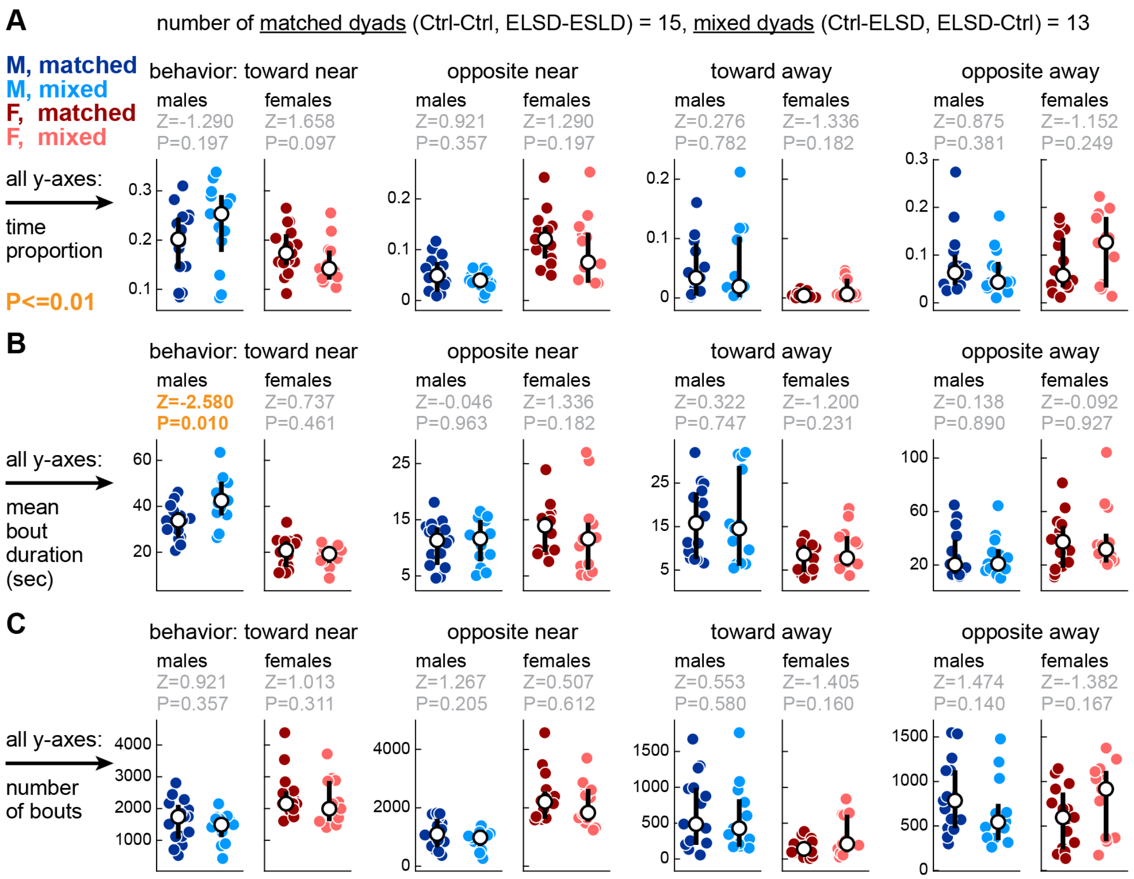

S2 Fig. Behavioral bout structure from Experiment 1 variables – focus on dyad type differences within prairie vole sexes. A. Time proportions (y-axes) for each behavior (four subplot columns) were calculated per individual animal (data points) and quantified for dyad type differences within sexes using median and interquartile ranges (black circles/bars), alongside Wilcoxon rank sum tests (Z and P values). Significant effects are highlighted in orange font. The four behavior categories – “toward near”, “opposite near”, “toward away”, “opposite away” – were defined by discretizing two continuous variables from Experiment 1: direction to divider and distance to divider, both derived from a keypoint tracker (DeepLabCut) as described in the main manuscript (see Methods and **Fig 5** description in the Results). **B-C.** Same layout, but showing mean bout duration and number of bouts on the y-axes. Only one significant effect was observed: “toward near” behavioral bouts lasted longer in males from mixed compared to matched dyads, supporting the time-series results in **Fig 2D** and the bout metrics in **S1 Fig**. No other differences between dyad types were found, unlike the exacerbation of sex differences in the main manuscript. This suggests that sex and dyad matching interact in quantifiable manners, which deserves further investigation in prairie voles and other species. ELSD: early-life sleep disruption. Ctrl: control. Underlying processed data and plotting code for this figure are available at figshare (https://doi.org/10.6084/m9.figshare.31820266).
