# Supplementary material for: Social compatibility in opposite-sex prairie vole pairs is modulated by early-life sleep experience
Source: PLoS Biol. 2026 Mar 27;24(3):e3003434. doi: 10.1371/journal.pbio.3003434 (PMC13043049; doi:10.1371/journal.pbio.3003434)
Supplement: S3 Fig — (PDF) [file pbio.3003434.s005.pdf]

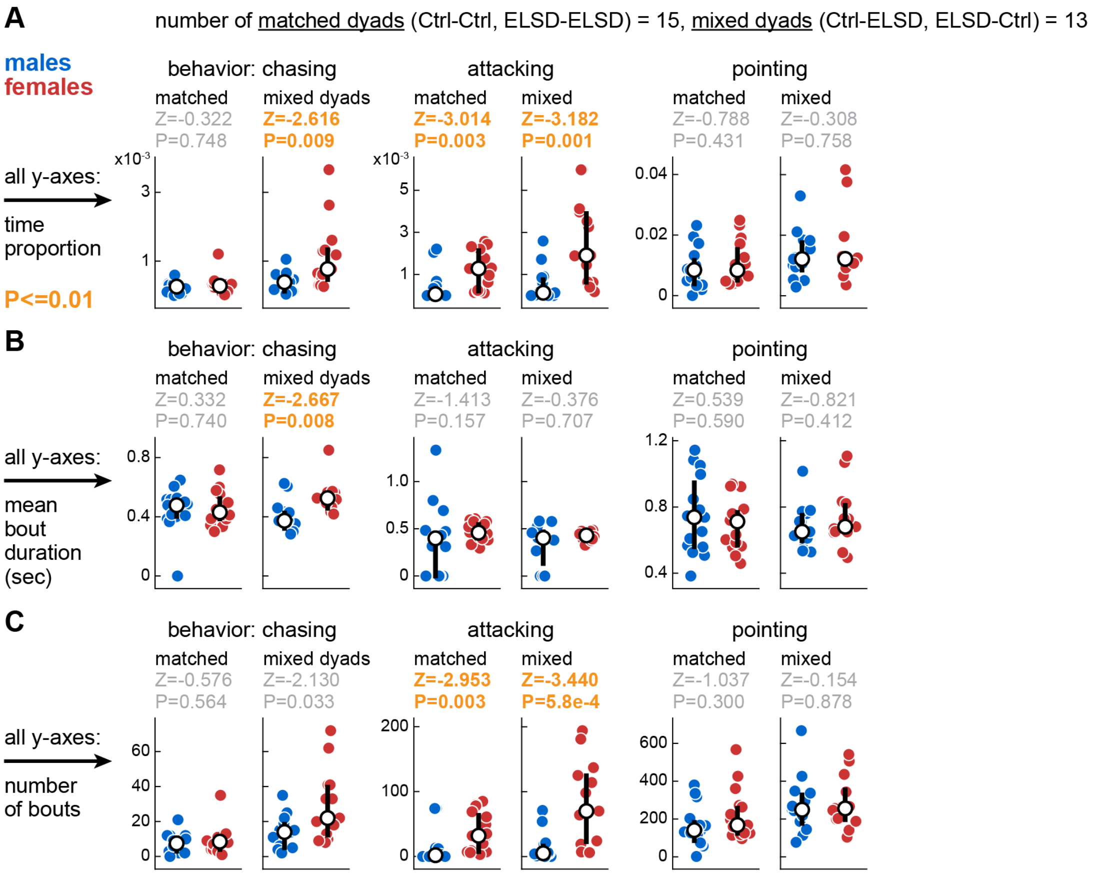

S3 Fig. Behavioral bout structure from Experiment 2 variables – focus on sex differences within matched and mixed prairie vole dyads. A. Time proportions (y-axes) for each behavior (three subplot columns) were calculated per individual animal (data points) and quantified for sex differences within dyad types using median and interquartile ranges (black circles/bars), alongside Wilcoxon rank sum tests (Z and P values). Significant effects are highlighted in orange font. The three social role categories – chasing, attacking, pointing – were annotated using a postural motif tracker (LabGym2) combined with supervision, as described in the main manuscript (see Methods). **B-C.** Same layout, but showing mean bout duration and number of bouts on the y-axes. Main takeaway: female-to-male aggression seems primarily explained by the number of bouts, more than bout duration, adding context to the time-series effects in **Fig 3B**. However, see **S4 Fig** for within-sex analyses, which revealed other effects regarding chasing behaviors. Other sex differences, or lack thereof, in this figure collectively show varying effects of dyad type, suggesting that the ethology of rodent dyad matching is multifaceted and highly amenable to quantification. ELSD: early-life sleep disruption. Ctrl: control. Underlying processed data and plotting code for this figure are available at figshare (https://doi.org/10.6084/m9.figshare.31820266).
