# Supplementary material for: Social compatibility in opposite-sex prairie vole pairs is modulated by early-life sleep experience
Source: PLoS Biol. 2026 Mar 27;24(3):e3003434. doi: 10.1371/journal.pbio.3003434 (PMC13043049; doi:10.1371/journal.pbio.3003434)
Supplement: S4 Fig — (PDF) [file pbio.3003434.s006.pdf]

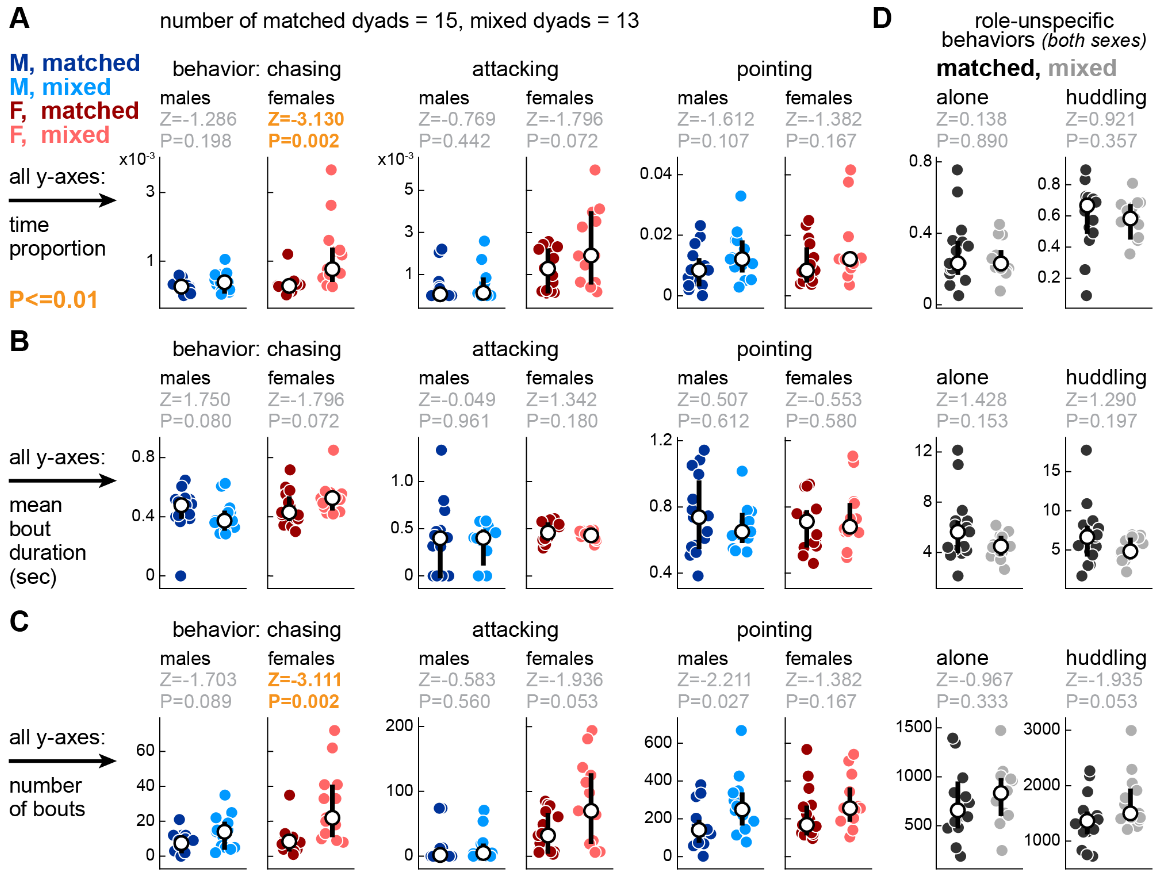

S4 Fig. Behavioral bout structure from Experiment 2 variables – focus on dyad type differences within prairie vole sexes. A. Time proportions (y-axes) for each behavior (three subplot columns) were calculated per individual animal (data points) and quantified for dyad type differences within sexes using median and interquartile ranges (black circles/bars), alongside Wilcoxon rank sum tests (Z and P values). Significant effects are highlighted in orange font. The three social role categories – chasing, attacking, pointing – were annotated using a postural motif tracker (LabGym2) combined with supervision, as described in the main manuscript (see Methods). **B-C.** Same layout, but showing mean bout duration and number of bouts on the y-axes. Only one significant effect was observed: bouts of female-to-male chasing behaviors were more frequent (but not longer-lasting) in mixed compared to matched dyads, providing context for the higher time percentage of female-to-male chasing in mixed dyads (this figure), as well as the time-series results in the main manuscript (**Fig 3D**) and the bout metrics in another supplement (**S3 Fig**). **D.** Similar analysis, but for mutual (role-unspecific) behaviors. The y-axes correspond to the subplot rows in panels **A-C**. Across all panels in this figure, no robust dyad type differences were found aside from those in the "chasing" column, unlike the exacerbation of sex differences in the main manuscript. This suggests that sex and dyad matching interact in quantifiable manners, which deserves further investigation in prairie voles and other species. ELSD: early-life sleep disruption. Ctrl: control. Underlying processed data and plotting code for this figure are available at figshare (https://doi.org/10.6084/m9.figshare.31820266).
