# Supplementary material for: Social compatibility in opposite-sex prairie vole pairs is modulated by early-life sleep experience
Source: PLoS Biol. 2026 Mar 27;24(3):e3003434. doi: 10.1371/journal.pbio.3003434 (PMC13043049; doi:10.1371/journal.pbio.3003434)
Supplement: S5 Fig — (PDF) [file pbio.3003434.s007.pdf]

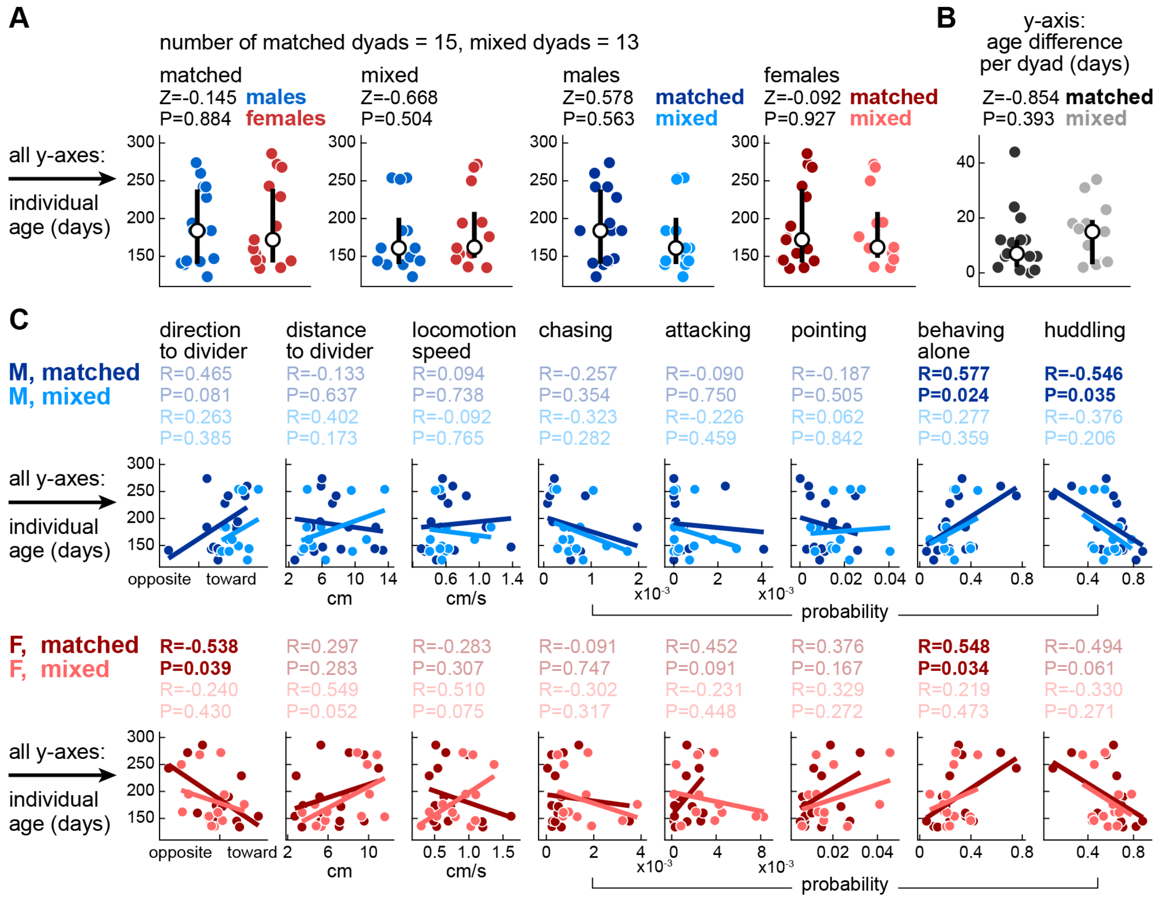

S5 Fig. Examining prairie vole age as a covariate: no evidence that age confounded our dyad type comparisons. A. Age distributions across groups. All y-axes depict age in days per individual animal (data points) along with median and interquartile ranges (black circles/bars). The two leftmost graphs compare sexes within each dyad type. The two rightmost graphs show the same data, but comparing dyad types within each sex. **B.** Same quantification methods but showing age difference within each dyad (data points). No significant effects were found in panels **A-B**, according to Wilcoxon rank sum tests (Z and P values), showing that age and age differences were evenly distributed across groups, i.e., there is no evidence of age biases in the dyad type comparisons. **C.** The y-axes again depict age per individual animal (data points), but this time relative to averaged behavioral variables from Experiments 1 and 2 (x-axes), forming scatterplots with linear fits and correlation coefficients. The behavioral variables on the x-axes are the same as the main correlation analyses (**Fig 4**). A few significant effects were observed, further differentiating dyad types. For example, older animals (regardless of sex) were more likely to behave alone during Experiment 2, but this effect was significant only in matched dyads. Prairie voles have indeed been shown to disperse and explore more as they age (McGuire et al., 1993 – Behav Ecol Sociobiol, doi: 10.1007/BF00183784; Milman et al., 2026 – Cereb Cortex, doi: 10.1093/cercor/bhaf321). This suggests that our experimental system replicated previously known behavioral tendencies in prairie voles, providing positive control for the dyad matching effects. However, these minor findings are beyond our scope and interpreted cautiously. The main point with this supplement is to report age distributions across sex/dyad groupings (panels **A-B**) alongside behavior-specific correlations for transparency and completeness (panel **C**). Underlying processed data and plotting code for this figure are available at figshare (https://doi.org/10.6084/m9.figshare.31820266).
