# Supplementary material for: Social compatibility in opposite-sex prairie vole pairs is modulated by early-life sleep experience
Source: PLoS Biol. 2026 Mar 27;24(3):e3003434. doi: 10.1371/journal.pbio.3003434 (PMC13043049; doi:10.1371/journal.pbio.3003434)
Supplement: S6 Fig — (PDF) [file pbio.3003434.s008.pdf]

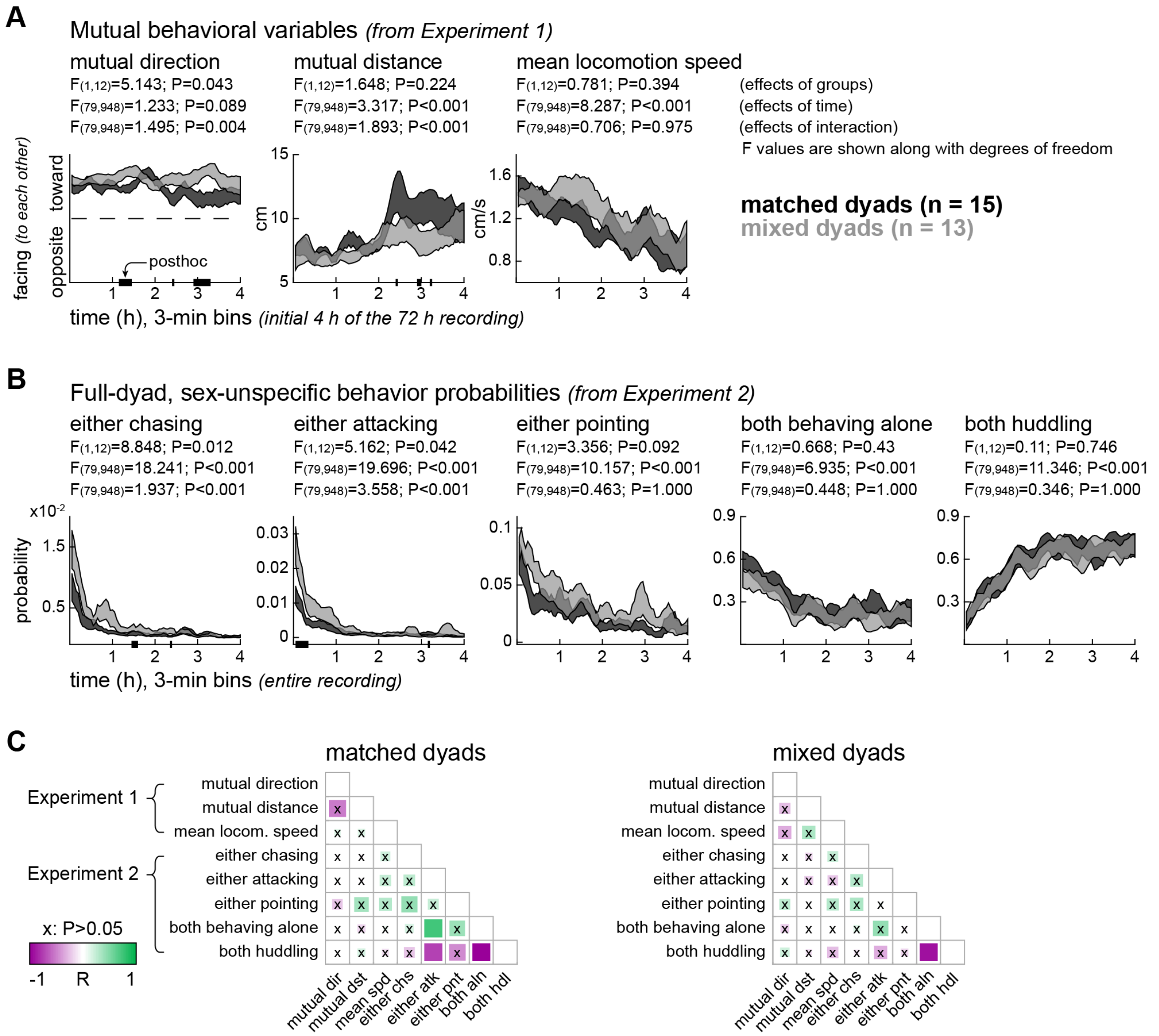

S6 Fig. Mutual and/or sex-unspecific behavioral variables in prairie voles, using dyads (not individuals) as the units for statistical variation. A. Three scales (y-axes) were created to supplement Experiment 1 (**Fig 2**). Mutual direction: a zero-centered scale (-1 to 1) ranging from both animals facing toward each other to both facing opposite from each other. Mutual distance: distance between the two animals regardless of the home cage divider. Mean locomotion speed: averaged from both animals in each dyad. The x-axes show a finer temporal resolution (3 min bins) than **Fig 2** (1 h bins), focusing on the initial 4 h of the 72-h recording, when animals were more active. The results in panel **A** further support dyad matching: mixed dyads exhibited a higher incidence of toward-each-other behaviors compared to matched dyads – an effect primarily driven by males, according to **Fig 2B-D**. Furthermore, mixed dyads showed lower mutual distance compared to matched dyads – an effect not captured in **Fig 2**, but consistent with the bout metrics in **S1-S2 Figs**, which show longer duration of behaviors “toward and near” the divider, especially in males. F statistics were obtained using two-way ANOVA with time bins as repeated measures. **B.** Similar, but from sex-unspecific behavior probabilities, supplementing Experiment 2 (**Fig 3**). The results also support the main manuscript: chasing and attacking behaviors were more frequent in mixed dyads – an effect primarily driven by females, according to **Fig 3B-D**. **C.** Correlation matrices like **Fig 4** but based on the variables analyzed here. Dyad types are further differentiated. Underlying processed data and plotting code for this figure are available at figshare (https://doi.org/10.6084/m9.figshare.31820266).
