# Supplementary material for: Social compatibility in opposite-sex prairie vole pairs is modulated by early-life sleep experience
Source: PLoS Biol. 2026 Mar 27;24(3):e3003434. doi: 10.1371/journal.pbio.3003434 (PMC13043049; doi:10.1371/journal.pbio.3003434)
Supplement: S7 Fig — (PDF) [file pbio.3003434.s009.pdf]

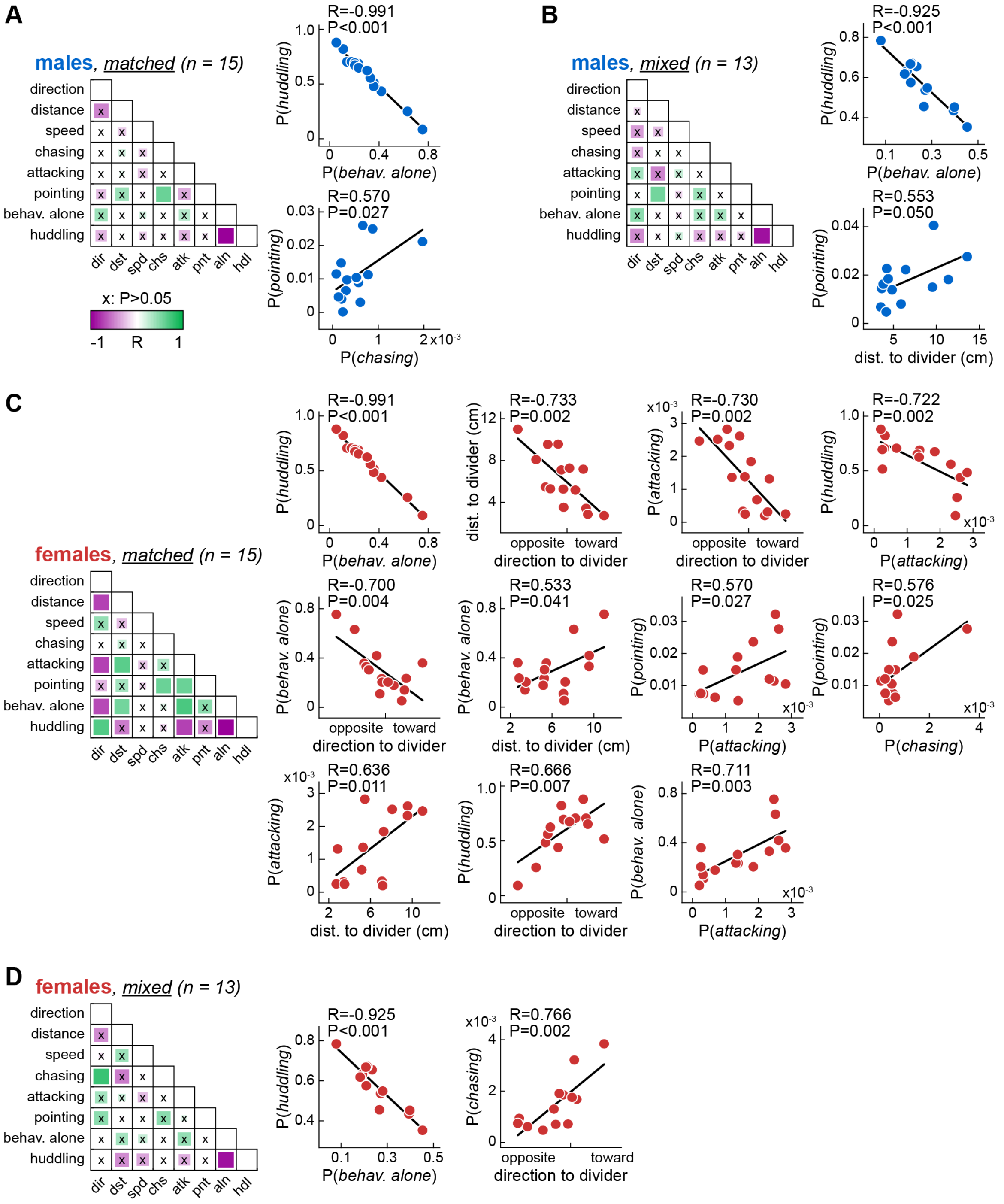

S7 Fig. Supplementary scatter plots for Fig 4. A-D. The panels show the same matrices as those in **Fig 4** but accompanied exclusively by scatter plots from significant correlations. **Fig 4** contains selected scatter plots (including non-significant ones) that are important to the main scope of this study. In that figure**,** the scatter plots are arranged in a structured layout to provide visual contrast for sex and dyad type differences. In this supplement, scatter plots from significant correlations are presented independently of any specific layout, for additional illustration. Underlying processed data and plotting code for this figure are available at figshare (https://doi.org/10.6084/m9.figshare.31820266).
