# Supplementary material for: Social compatibility in opposite-sex prairie vole pairs is modulated by early-life sleep experience
Source: PLoS Biol. 2026 Mar 27;24(3):e3003434. doi: 10.1371/journal.pbio.3003434 (PMC13043049; doi:10.1371/journal.pbio.3003434)
Supplement: S8 Fig — (PDF) [file pbio.3003434.s010.pdf]

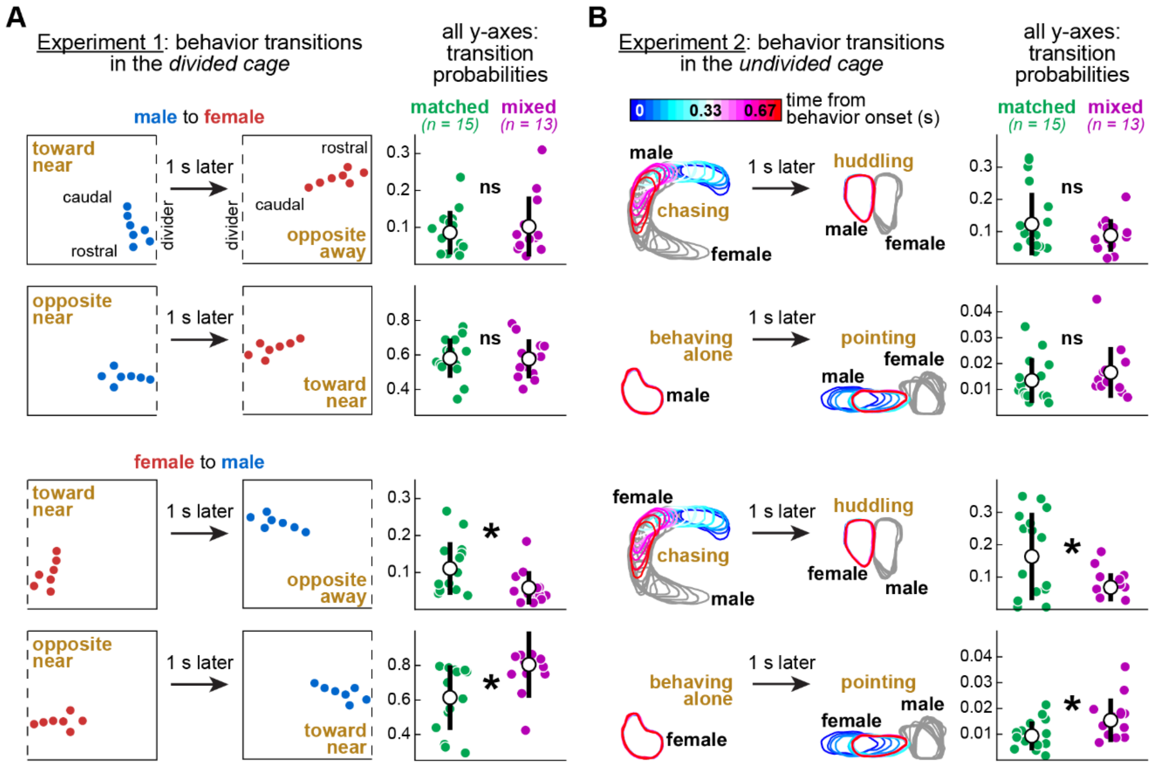

S8 Fig. Illustration of male-to-female and female-to-male behavior transitions in 1 s bins – supplementing Fig 5. A. Experiment 1: divided-cage cohabitation analyzed using keypoint tracking (DeepLabCut). Schematics for behavior transitions are shown on the left, and corresponding probability data (same as **Fig 5**) are shown on the right. The same selected transitions are shown for male-to-female and female-to-male, facilitating comparisons (see y-axes on top vs. bottom scatter plots). Significant differences between dyad types were confined to female-to-male transitions, suggesting that dyad matching and sex directionality jointly affect dyadic interactions on the timescale of seconds. **B.** Experiment 2, undivided-cage cohabitation, analyzed using social role tracking (LabGym2). Same layout as panel **A**, again evidencing that both dyad matching and sex directionality affect dyadic behaviors. Apart from the nuances of each specific transition, the main conclusion from **Fig 5** and this supplement is that dyad matching and sex directionality effects emerged across different recording paradigms, suggesting robust underlying mechanisms that deserve further investigation in prairie voles and other species. See **Table 3** for main statistical effects, and **Fig 5** for a comprehensive illustration of behavior transitions using directed graphs. Underlying processed data and plotting code for this figure are available at figshare (https://doi.org/10.6084/m9.figshare.31820266).
